# Loss of pyruvate kinase M2 limits growth and triggers innate immune signaling in endothelial cells

**DOI:** 10.1038/s41467-018-06406-8

**Published:** 2018-10-09

**Authors:** Oliver A. Stone, Mohamed El-Brolosy, Kerstin Wilhelm, Xiaojing Liu, Ana M. Romão, Elisabetta Grillo, Jason K. H. Lai, Stefan Günther, Sylvia Jeratsch, Carsten Kuenne, I-Ching Lee, Thomas Braun, Massimo M. Santoro, Jason W. Locasale, Michael Potente, Didier Y. R. Stainier

**Affiliations:** 1 Department of Developmental Genetics, Max Planck Institute for Heart and Lung Research, Ludwigstrasse 43, 61231 Bad Nauheim, Germany; 2 Angiogenesis & Metabolism Laboratory, Max Planck Institute for Heart and Lung Research, Ludwigstrasse 43, 61231 Bad Nauheim, Germany; 3 Department of Pharmacology and Cancer Biology, Duke University School of Medicine, Durham, NC 27710 USA; 4 Department of Cardiac Development and Remodelling, Max Planck Institute for Heart and Lung Research, Ludwigstrasse 43, 61231 Bad Nauheim, Germany; 5 Department of Oncology, KUL, Herestraat 49, 3000 Leuven, Belgium; 6 ECCPS Bioinformatics and Deep Sequencing Platform, Max Planck Institute for Heart and Lung Research, Ludwigstrasse 43, 61231 Bad Nauheim, Germany; 7 Biomolecular Mass Spectrometry, Max Planck Institute for Heart and Lung Research, Ludwigstrasse 43, 61231 Bad Nauheim, Germany; 8 Department of Biology, University of Padua, Viale Giuseppe Colombo 3, 10141 Padua, Italy; 9 Present Address: Department of Physiology, Anatomy and Genetics, BHF Centre of Research Excellence, University of Oxford, Oxford, OX1 3PT UK; 10 Present Address: Mechanobiology Institute, National University of Singapore, Singapore, 117411 Singapore

## Abstract

Despite their inherent proximity to circulating oxygen and nutrients, endothelial cells (ECs) oxidize only a minor fraction of glucose in mitochondria, a metabolic specialization that is poorly understood. Here we show that the glycolytic enzyme pyruvate kinase M2 (PKM2) limits glucose oxidation, and maintains the growth and epigenetic state of ECs. We find that loss of PKM2 alters mitochondrial substrate utilization and impairs EC proliferation and migration in vivo. Mechanistically, we show that the NF-κB transcription factor RELB is responsive to PKM2 loss, limiting EC growth through the regulation of P53. Furthermore, S-adenosylmethionine synthesis is impaired in the absence of PKM2, resulting in DNA hypomethylation, de-repression of endogenous retroviral elements (ERVs) and activation of antiviral innate immune signalling. This work reveals the metabolic and functional consequences of glucose oxidation in the endothelium, highlights the importance of PKM2 for endothelial growth and links metabolic dysfunction with autoimmune activation in ECs.

## Abstract (web summary)

The glycolytic enzyme pyruvate kinase M2 (PKM2) is required for nucleotide synthesis and cell proliferation. Using gene expression and metabolomics analyses, the authors here show that PKM2 regulates methionine metabolism and DNA methylation in endothelial cells.

## Introduction

The vertebrate vascular system comprises a vast and evolutionarily conserved network that supports tissue growth and homoeostasis^1^. Recent work has highlighted the high glycolytic rate of the endothelial cells (ECs) that line this network^2^, which under physiological conditions metabolize almost 90% of cellular glucose anaerobically to produce lactate^3^. The ability to generate ATP in this manner permits EC migration into non-perfused tissues and allows the expansion of vascular networks during organ growth^2,4,5^. Given these distinct metabolic traits, endothelial mitochondria have been considered to function largely as signalling organelles^6,7^, and the consequences of enhanced glucose oxidation in ECs are not well described. Recent work has shown that mitochondria play fundamental roles in EC growth and homoeostasis; β-oxidation of fatty acids is required for dNTP synthesis^8^, while glutamine metabolism is an essential source of TCA cycle intermediates that are necessary to support macromolecule biosynthesis in ECs^3,9^.

To gain insight into the functional and metabolic consequences of glucose oxidation in ECs, we analysed the function of the pyruvate kinase (PK) isozyme PKM2, which has been associated with aerobic glycolysis and growth in cancer cells^10,11^, and the maintenance of mitochondrial function in diabetic nephropathy^12^. Pyruvate kinase catalyses the final step in glycolysis, generating pyruvate and ATP from phosphoenolpyruvate and ADP^13^. In higher vertebrates, two genes (*PKLR* and *PKM*) encode four PK isozymes that vary in their expression patterns and activity^14^. Alternative splicing of the *PKM* gene to include exon 9 or 10 generates the PKM1 and PKM2 isozymes, respectively^15^. While PKM1 exhibits constitutively high PK activity and its expression is associated with a decrease in cell growth, the catalytic activity of PKM2 is modulated allosterically by critical metabolic intermediates^16–18^ and post-translationally in response to growth factor signalling and reactive oxygen species (ROS)^10,19^, providing a focal point for the integration of cellular signalling and redox status with glycolytic flux. Here we show that loss of PKM2 in ECs results in TCA cycle dysfunction, cell cycle arrest and the induction of viral mimicry by endogenous retroviral transcripts.

## Results

### Loss of endothelial PKM2 alters mitochondrial metabolism

**Fig. 1:**
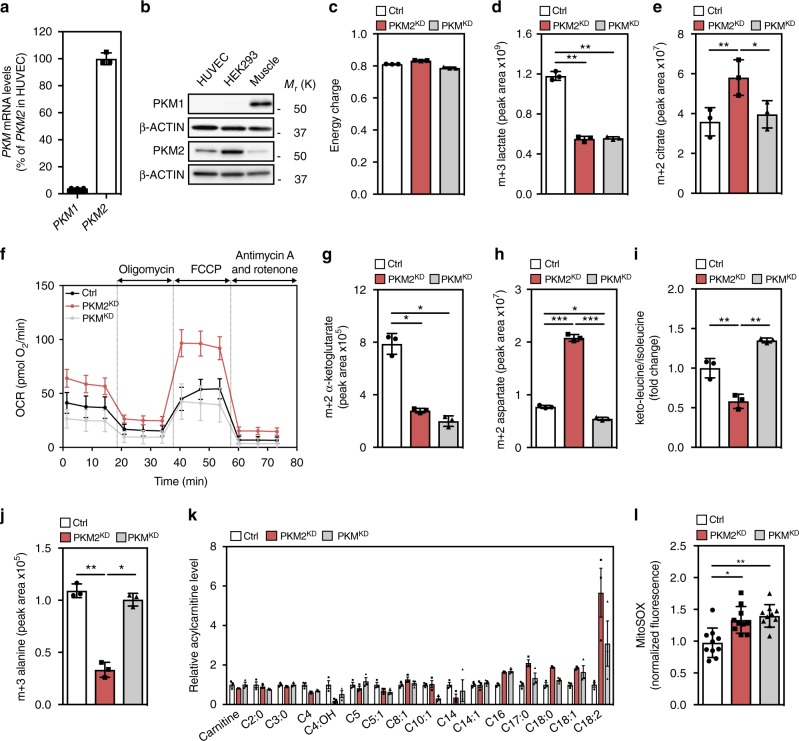
Loss of endothelial PKM2 leads to mitochondrial dysfunction. **a** RT-qPCR analysis of *PKM* mRNA expression showing that *PKM2* is more abundantly expressed in HUVECs (*n* = 3). **b** Western blot analysis of PKM1 and PKM2 expression in HUVECs, HEK293 and mouse skeletal muscle. **c** Relative energy charge ([ATP] + 1/2[ADP]/[ATP] + [ADP] + [AMP]) in control, PKM2^KD^ and PKM^KD^ ECs (*n* = 3). Incorporation of [U-13C_6_]-glucose into m+3 lactate (**d**) and m+2 citrate (**e**) in control, PKM2^KD^ and PKM^KD^ ECs (*n* = 3). **f** Oxygen consumption rate (OCR) in control, PKM2^KD^ and PKM^KD^ ECs under basal conditions and in response to oligomycin, fluoro-carbonyl cyanide phenyl-hydrazone (FCCP) and antimycin A/rotenone (*n* = 6, data represent mean ± s.d.). **g** Incorporation of [U-13C_6_]-glucose into m+2 α-ketoglutarate in control, PKM2^KD^ and PKM^KD^ ECs (*n* = 3). **h** Incorporation of [U-13C_6_]-glucose into m+2 aspartate in control, PKM2^KD^ and PKM^KD^ ECs (*n* = 3). **i** Ratio of keto-leucine/isoleucine in control, PKM2^KD^ and PKM^KD^ ECs. **j** Incorporation of [U-13C_6_]-glucose into m+3 alanine in control, PKM2^KD^ and PKM^KD^ ECs (*n* = 3). **k** Relative levels of different acylcarnitine species in control, PKM2^KD^ and PKM^KD^ ECs (*n* = 3). **l** Normalized MitoSOX fluorescence intensity in control, PKM2^KD^ and PKM^KD^ ECs. **a**, **c**–**l** Data represent means ± s.d., m+*n* represents the mass isotopomers for individual metabolites (***P < 0.001, **P < 0.01, *P < 0.05 by one-way analysis of variance (ANOVA) followed by Tukey’s HSD test)

The *PKM* gene is abundantly expressed in ECs (Supplementary Figure 1a), and RT-qPCR (Fig. 1a) and western blot (Fig. 1b) analyses show that PKM2 is the predominant isoform. To determine the function of endothelial PKM2, we first assessed the efficacy of validated siRNA duplexes targeting specifically PKM2 (PKM2^KD^) or both PKM splice isoforms (PKM^KD^) in primary human umbilical vein ECs (HUVECs) (Supplementary Figure 1)^20^. Functionally, while neither PKM2^KD^ nor PKM^KD^ significantly changed cellular energy charge (Fig. 1c), incorporation of [U-13C_6_]-glucose-derived carbon into m+3 lactate (Fig. 1d) and extracellular acidification rate (ECAR) (Supplementary Figure 1g) were significantly reduced in both conditions. The reduction in labelled lactate was coupled to an increase in [U-13C_6_]-glucose labelling of citrate (Fig. 1e) and oxygen consumption rate (OCR) (Fig. 1f) in PKM2^KD^ ECs but not in PKM^KD^ ECs, indicating that PKM isoform expression is a critical determinant of the fate of glucose-derived carbon in ECs. Intriguingly, analysis of steady-state levels of TCA cycle intermediates revealed a decrease in total α-ketoglutarate (α-KG), fumarate and malate in PKM2^KD^ and PKM^KD^ ECs, while aspartate levels were significantly increased only in PKM2^KD^ ECs (Supplementary Figure 1h). Furthermore, [U-13C_6_]-glucose labelling of α-KG (Fig. 1g) was significantly reduced in PKM2^KD^ and PKM^KD^ ECs, while aspartate labelling was increased only in PKM2^KD^ ECs (Fig. 1h), indicating that significant changes to TCA cycle metabolism occur in the absence of PKM2. Glycolysis is not the sole carbon source for the TCA cycle, which is also fuelled by branched chain amino acid (BCAA) oxidation, pyruvate anaplerosis, fatty acid oxidation (FAO) and glutamine metabolism^3,9^. We found that PKM2^KD^, but not PKM^KD^, led to a reduction in the ratio of keto-leucine to isoleucine (Fig. 1i) and [U-13C_6_]-glucose labelling of m+3 alanine (Fig. 1j), while an accumulation of long-chain acylcarnitines (Fig. 1k), and reduced m+3 malate (Supplementary Figure 1i) were observed in both PKM2^KD^ and PKM^KD^ ECs. These data indicate that BCAA oxidation, pyruvate anaplerosis and FAO are impaired in PKM2^KD^ ECs. Mitochondria are an important source of ROS in endothelial cells, where at low levels ROS perform critical signalling functions^21^. We found that while the global ratio of GSSG/GSH (Supplementary Figure 1j) and cellular ROS levels (Supplementary Figure 1k) were not changed in PKM2^KD^ ECs, there was an increase in mitochondrial superoxide levels (Fig. 1l). Furthermore, we did not observe changes in mitochondrial DNA content (Supplementary Figure 1l). Collectively, these data show that loss of PKM2 alters mitochondrial metabolism and is associated with the disruption of multiple pathways that fuel TCA cycle metabolism and cell growth.

### Loss of PKM2 impairs angiogenic sprouting

**Fig. 2:**
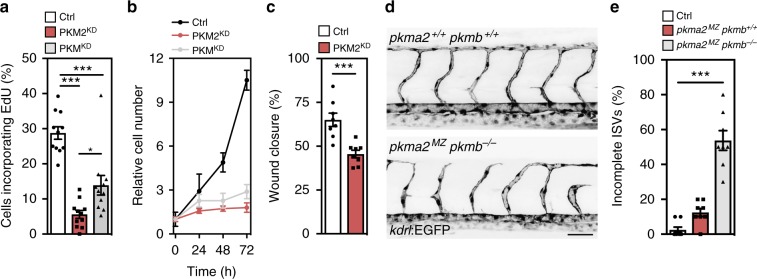
Loss of PKM2 leads to angiogenic sprouting defects. **a** EdU-positive cell numbers in control, PKM2^KD^ and PKM^KD^ ECs (*n* = 11). **b** Relative cell numbers at the indicated time points in control, PKM2^KD^ and PKM^KD^ ECs (*n* = 6). **c** Scratch wound assay in control and PKM2^KD^ ECs (*n* = 8). **d** Representative confocal projections of 48 hpf *Tg(kdrl:EGFP) pkma2*^*+/+*^ *pkmb*^*+/+*^ and *pkma2*^*MZ*^ *pkmb*^*−/−*^ zebrafish embryos (scale bar = 50 μm). **e** Percentage of intersegmental vessels (ISVs) that have failed to connect with neighbouring ISVs in 48 hpf zebrafish embryos (*n* = 8). Data in **a**–**c** and **e** represent means ± s.e.m. (***P < 0.001 by one-way analysis of variance (ANOVA) followed by Tukey’s HSD test (**a**, **e**) or two-tailed Student's *t* test (**c**))

To understand the functional importance of PKM2 for angiogenic sprouting, we assessed EC migration and proliferation in primary cells, zebrafish and mice. In HUVECs, PKM2^KD^ led to a decrease in the number of 5-ethynyl-2′-deoxyuridine positive (EdU^+^) (Fig. 2a) and phospho-Histone H3 positive (pHH3^+^) (Supplementary Figure 2a) ECs, and cell cycle arrest in G_0_/G_1_ at 48 h after siRNA transfection (Supplementary Figure 2b), resulting in a significant reduction in relative EC number (Fig. 2b). We also observed a decrease in EC proliferation using ML265, a small-molecule activator of PKM2 tetramerization that mimics the effects of PKM2 loss of function, providing further evidence that modulation of PKM2 activity can inhibit EC proliferation (Supplementary Figure 2c). Furthermore, PKM2^KD^ significantly impaired wound closure in an in vitro scratch wound assay, indicating that PKM2 may also play an important role in EC migration (Fig. 2c). To assess the in vivo relevance of these findings, we generated mutants in the zebrafish orthologues, *pkma* and *pkmb* (Supplementary Figure 2d–g). The zebrafish *pkma* gene displays a similar genomic organization as its mammalian orthologues (Supplementary Figure 2d), and is alternatively spliced into exon 9 or 10 to give rise to mRNAs that we have designated as *pkma1* and *pkma2*. In contrast, in zebrafish and other teleosts, the *pkmb* gene contains 11 exons and is not subject to alternative splicing (Supplementary Figure 2d). Clustering analysis based on a 56 amino acid sequence with homology to the amino acids encoded by exon 9 or 10 of the human *PKM* gene (Supplementary Figure 2e), indicates that *pkmb* is an orthologue of mammalian *PKM2*, not *PKM1*. Confocal analyses of wild-type, maternal zygotic *pkma2* (*pkma2*^*mz*^) and *pkma2*^*mz*^*/pkmb*^*−/−*^ mutant embryos revealed defects in the formation of the intersegmental vessels (ISVs) (Fig. 2d). Live imaging demonstrated impaired proliferation and migration of ECs in the ISVs of *pkma2*^*mz*^*/pkmb*^*−/−*^ mutants (Supplementary Movie 1). Quantification of incomplete ISVs at 48 h post fertilization (hpf) reveals a significant increase in the number of ISVs that fail to make connections with their neighbours in *pkma2*^*mz*^*/pkmb*^*−/−*^ mutants (Fig. 2e), indicating that Pkm2 regulates blood vessel growth in vivo.

**Fig. 3:**
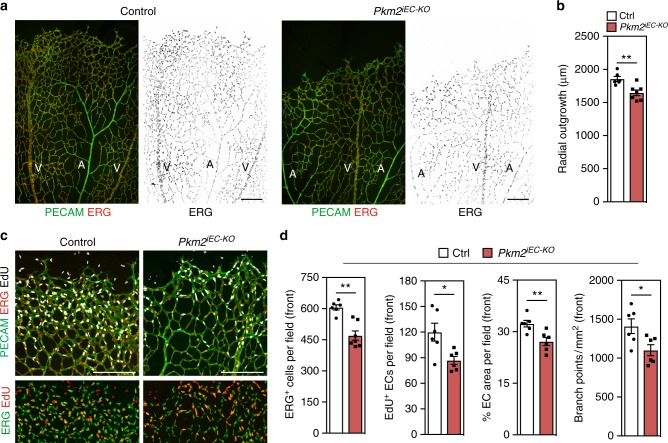
Acute deletion of *Pkm2* in mouse endothelial cells leads to angiogenic sprouting defects. **a** Representative confocal projections of PECAM, ERG and EdU immunostained retinas from control and *Pkm2*^*iEC-KO*^ mice (A, Artery; V, Vein). **b** Radial outgrowth in control and *Pkm2*^*iEC-KO*^ mice (*n* = 6). **c** Representative confocal projections of angiogenic front vessels in PECAM, ERG and EdU-stained retinas from control and *Pkm2*^*iEC-KO*^ mice. **d** ERG^+^ ECs, EdU^+^ ECs, EC area per field and branch point density at the angiogenic front of control and *Pkm2*^*iEC-KO*^ mice (*n* = 6). All analyses in the mouse retina were performed on postnatal day 7. Data in **b** and **d** represent means ± s.e.m. (**P < 0.01, *P < 0.05 by two-tailed Student's *t* test). Scale bars in **a** and **c** = 200 μm

To interrogate the endothelial cell autonomous requirement for PKM2 during vessel growth, we conditionally deleted exon 10 of the *Pkm* gene (Supplementary Figure 3a, b) in endothelial cells of mice and analysed angiogenesis in the postnatal retina. To this end, we intercrossed *Pkm2*^*flox*^ mice with *Pdgfb-Cre*^*ERT2*^ mice (referred to hereafter as *Pkm2*^*iEC-KO*^) and induced recombination by 4-hydroxy-tamoxifen (4-OHT) injection from postnatal day (P) 1–3. Staining of retinas for PECAM (marking EC membranes) and ERG (marking EC nuclei) at P7 revealed reduced outgrowth of a hypo-cellular vascular plexus in *Pkm2*^*iEC-KO*^ mice (Fig. 3a, b). To determine the cause of this phenotype, we analysed the morphology and growth of vessels at the angiogenic front in PECAM, ERG and EdU-stained retinas (Fig. 3c). Quantification revealed a significant reduction in EC number, proliferation, vessel area and branching in *Pkm2*^*iEC-KO*^ mice (Fig. 3d). In contrast to the vascular defects at the angiogenic front, the central plexus of the retina was less affected by PKM2 inactivation using the *Pdgfb-Cre*^*ERT2*^ (Supplementary Figure 3c). While endothelial cellularity was significantly reduced, vessel area and branch point density were similar to controls (Supplementary Figure 3e). These findings are consistent with the enrichment of PKM2 in angiogenic ECs^22^ and suggest that PKM2 signalling is particularly important for vessel sprouting at the growing angiogenic front. To assess the possible contribution of vessel regression, we quantified the number of empty collagen IV^+^ sleeves and observed no significant difference between control and *Pkm2*^*iEC-KO*^ mice (Supplementary Figure 3d, e). Taken together, these data suggest that PKM2 is a crucial regulator of endothelial migration and proliferation, whose inactivation compromises vascular expansion.

### Coordinated metabolic and protein changes following PKM2 loss

**Fig. 4:**
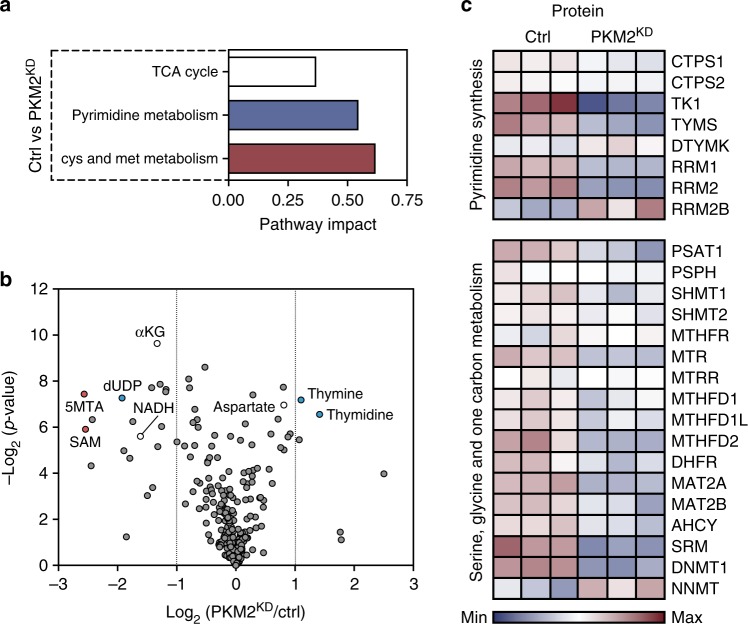
Loss of PKM2 leads to coordinated metabolic and protein expression changes. **a** Analysis of steady-state metabolite levels in control vs PKM2^KD^ ECs identifies the TCA cycle, pyrimidine metabolism and cysteine and methionine metabolism as significantly regulated pathways. **b** Metabolomics profile of control vs PKM2^KD^ ECs (log_2_(fold change) vs −log_2_(*p* value), two-tailed Student's *t* test, *n* = 3). **c** Heat map of protein expression for key enzymes in the pyrimidine synthesis and serine, glycine, one-carbon metabolic networks in control vs PKM2^KD^ ECs

To gain further insight into the global metabolic changes associated with loss of endothelial PKM2, we performed pathway analyses of steady-state metabolites and identified pathways linked to mitochondrial metabolism^23,24^, including TCA cycle, pyrimidine synthesis and cysteine and methionine metabolism (Fig. 4a, specific metabolites highlighted in Fig. 4b). Deletion of *PKM2* in primary mouse embryonic fibroblasts (MEFs) leads to impaired pyrimidine synthesis that does not correlate with gene expression changes^25^. To determine if loss of PKM2 is associated with gene expression changes in ECs, we performed a proteomics analysis that revealed changes to the expression of a number of key enzymes in the pyrimidine synthesis, and serine, glycine and one-carbon metabolism (SGOC) pathways (Fig. 4c and Supplementary Figure 4a). These data indicate that the metabolic changes observed in PKM2^KD^ ECs (Fig. 4a, b) correlate with protein-level changes in the key enzymes controlling these pathways, leading us to hypothesize that there may be a coordinated transcriptional response to the loss of PKM2 in ECs.

### RELB-P53 suppresses endothelial growth in the absence of PKM2

**Fig. 5:**
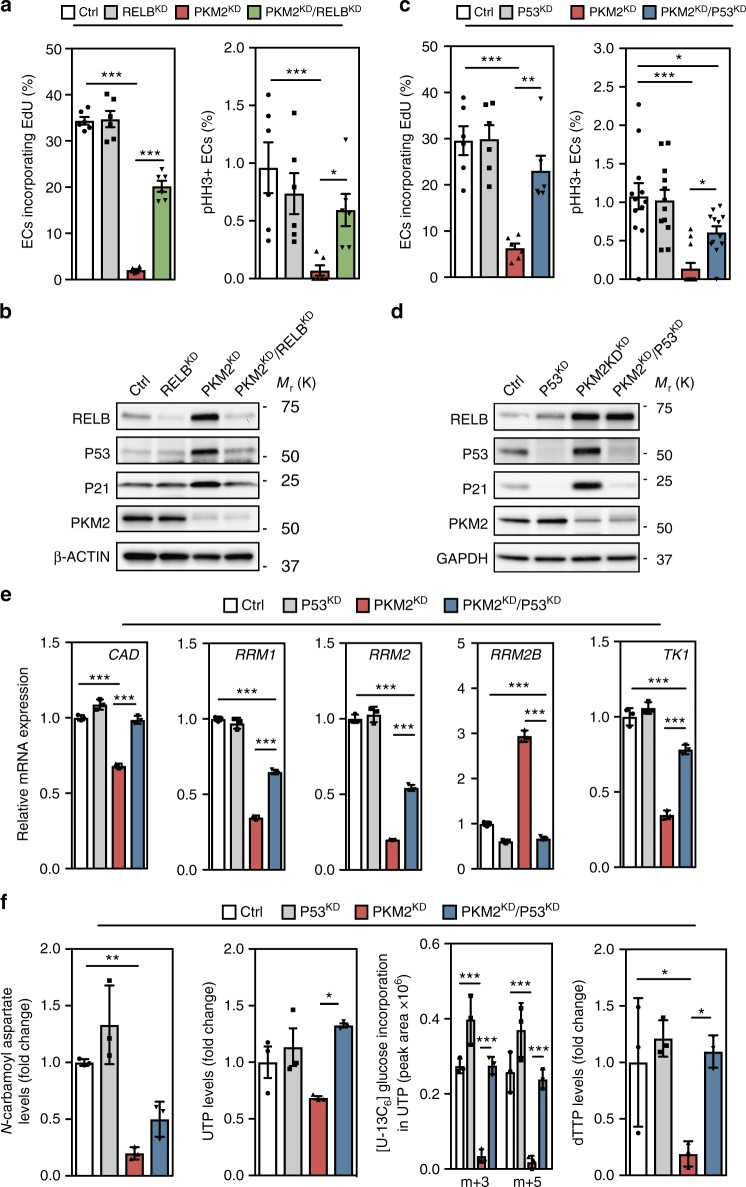
RELB-P53 suppresses endothelial growth in the absence of PKM2. **a** EdU and phospho-Histone H3 positive cell numbers in control, RELB^KD^, PKM2^KD^ and PKM2^KD^/RELB^KD^ ECs (*n* = 6). **b** Western blot analysis of RELB, P53, P21 and PKM2 expression in control, RELB^KD^, PKM2^KD^ and PKM2^KD^/RELB^KD^ ECs. **c** EdU and phospho-Histone H3 positive cell numbers in control, P53^KD^, PKM2^KD^ and PKM2^KD^/P53^KD^ ECs (*n* = 12). **d** Western blot analysis of RELB, P53, P21 and PKM2 expression in control, P53^KD^, PKM2^KD^ and PKM2^KD^/P53^KD^ ECs. **e** mRNA expression levels of key enzymes for pyrimidine synthesis in control, P53^KD^, PKM2^KD^ and PKM2^KD^/P53^KD^ ECs (RNA-seq analysis, *n* = 3). **f** Relative *N*-carbamoyl aspartate levels, UTP levels, incorporation of [U-13C_6_]-glucose into m+3 and m+5 UTP, and relative dTTP levels in control, P53^KD^, PKM2^KD^ and PKM2^KD^/P53^KD^ ECs (*n* = 3). **a**, **c** Data represent means ± s.e.m., **e**, **f** data represent means ± s.d., m+*n* represents the mass isotopomers for individual metabolites (***P < 0.001, **P < 0.01, *P < 0.05 by one-way analysis of variance (ANOVA) followed by Tukey’s HSD test)

Further analyses revealed that components of the NF-κB and TP53 transcription factor pathways, which have previously been linked with the regulation of metabolism^26–29^, are upregulated in PKM2^KD^ ECs (Supplementary Figure 5a, b). We thus set out to test if these pathways regulate EC growth in the absence of PKM2. Co-transfection of siRNA duplexes targeting *PKM2* and *REL*, *RELA* or *RELB*, revealed that silencing *RELB* significantly rescued the number of EdU^+^ (Fig. 5a and Supplementary Figure 5c) and pHH3^+^ (Fig. 5a) ECs, indicating that RELB mediates the cell cycle arrest observed in the absence of PKM2. Western blot analysis of control, RELB^KD^, PKM2^KD^ and PKM2^KD^/RELB^KD^ ECs revealed that RELB expression was increased in the absence of PKM2 and acts to induce the expression of P53 and its downstream target P21 (Fig. 5b).

Given the established role of the P53–P21 pathway in the regulation of cell cycle progression^30^, we hypothesized that RELB-mediated induction of P53–P21 might be the cause of the cell cycle arrest observed in PKM2^KD^ ECs. Quantification of EdU and phospho-Histone H3 (Fig. 5c) positive ECs, and total cell numbers (Supplementary Figure 5d) revealed a significant rescue of the cell cycle arrest observed in PKM2^KD^ ECs following P53 silencing. Furthermore, western blot analysis showed that the increase in P53 and its transcriptional target P21 in PKM2^KD^ ECs was abolished by co-transfecting siRNA duplexes targeting *P53*, while RELB expression was unchanged (Fig. 5d). These data show that RELB acts upstream of P53–P21 to induce cell cycle arrest in the absence of PKM2. In addition to its well-documented tumour suppressor functions^30^, there is an increasing appreciation of the importance of P53 in the regulation of metabolism in response to various types of cellular stress^26^. In tumour cells deprived of exogenous serine, P53 promotes glutathione synthesis at the expense of nucleotides to promote cell survival^31^. To identify potential transcriptional and metabolic changes controlled by P53, we performed RNA-seq and metabolomics analyses in control, P53^KD^, PKM2^KD^ and PKM2^KD^/P53^KD^ ECs. Analysis of the transcriptional response (Supplementary Figure 5e) revealed that P53 regulates the expression of key enzymes in the pyrimidine synthesis pathway (Fig. 5e). Volcano plots displaying metabolite profiles show that the relative levels of a number of metabolites in the pyrimidine synthesis pathway are reduced following PKM2^KD^ (Supplementary Figure 5f), and that silencing of P53 leads to a relative normalization of the levels of these metabolites (Supplementary Figure 5g). Comprehensive analysis of nucleotide levels revealed that PKM2^KD^ reduced the relative levels of *N*-carbamoyl aspartate, UTP and dTTP, and impaired incorporation of glucose-derived carbon into UTP, while silencing of P53 increased the levels of these metabolites (Fig. 5f). Collectively, these data indicate that a P53-dependent transcriptional programme controls the reduction in pyrimidine nucleotide levels in ECs lacking PKM2.

### Loss of PKM2 impairs methylation capacity and DNA methylation

**Fig. 6:**
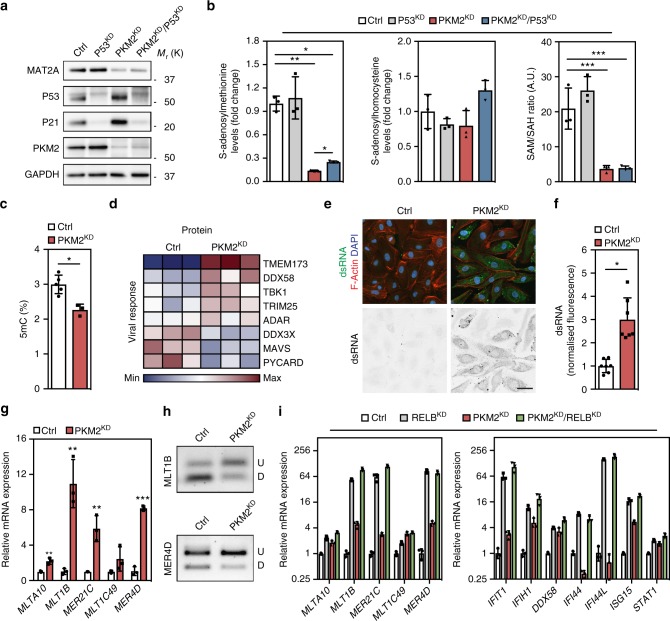
Loss of PKM2 impairs methylation capacity, reduces DNA methylation and leads to the expression of endogenous retroviral elements. **a** Western blot analysis of MAT2A, P53, P21 and PKM2 in control, P53^KD^, PKM2^KD^ and PKM2^KD^/P53^KD^ ECs. **b** Relative levels of S-adenosylmethionine and S-adenosylhomocysteine and SAM/SAH ratio in control, P53^KD^, PKM2^KD^ and PKM2^KD^/P53^KD^ ECs (*n* = 3). **c** Percentage 5-methylcytosine (5mC) levels in control and PKM2^KD^ ECs (*n* = 4). **d** Heat map of protein expression for components of the cellular response to viral infection in control and PKM2^KD^ ECs. **e** Representative confocal projections of total double-stranded RNA (dsRNA) staining in control and PKM2^KD^ ECs (scale bar = 20 μm). **f** Normalized fluorescence intensity for dsRNA staining in control and PKM2^KD^ ECs (*n* = 7). **g** Relative mRNA expression of the indicated endogenous retroviruses in control and PKM2^KD^ ECs (*n* = 3). **h** Restriction digestion of bisulfite-treated DNA amplified from the *MLT1B* and *MER4D* genomic loci from control and PKM2^KD^ ECs (U, undigested/unmethylated DNA; D, digested/methylated DNA). **i** Relative mRNA expression of the indicated endogenous retroviruses and interferon-stimulated genes in control, RELB^KD^, PKM2^KD^ and PKM2^KD^/RELB^KD^ ECs. **b**, **c**, **f**, **g**, **i** Data represent means ± s.d. (***P < 0.001, **P < 0.01, *P < 0.05 by one-way analysis of variance (ANOVA) followed by Tukey’s HSD test)

In addition to changes in the pyrimidine synthesis pathway, loss of PKM2 also led to significant changes in the steady-state levels of the one-carbon metabolite s-adenosylmethionine (SAM) (Fig. 4b), which were coupled to reduced expression of enzymes in the SGOC network (Fig. 4c). As the primary cellular methyl group donor, the abundance of SAM determines the methylation status of a broad range of substrates, including DNA and histones^32^. SAM is an intermediate metabolite in a wider metabolic network that encompasses the folate and methionine cycles, termed one-carbon metabolism^24^, and is synthesized from ATP and methionine, by methionine adenosyltransferases (MAT). MAT1A expression is restricted to the liver, while MAT2A and MAT2B are expressed in non-hepatic tissues and their expression levels have been shown to positively correlate with DNA methylation^33^. Western blot analysis of control, P53^KD^, PKM2^KD^ and PKM2^KD^/P53^KD^ ECs revealed a P53-independent reduction in the expression of MAT2A in the absence of PKM2 (Fig. 6a). Metabolomic analyses of control, P53^KD^, PKM2^KD^ and PKM2^KD^/P53^KD^ ECs showed a significant reduction in the steady-state levels of SAM (Fig. 6b), with no concomitant decrease in S-adenosylhomocysteine (SAH) in PKM2^KD^ and PKM2^KD^/P53^KD^ ECs (Fig. 6b), leading to a P53-independent drop in the ratio of SAM:SAH (Fig. 6b). These changes were associated with a reduction in the SAM-derived metabolite S-methyl-5-thioadenosine (5MTA) in PKM2^KD^ and PKM2^KD^/P53^KD^ ECs, with no change in methionine levels or GSH/GSSG ratio (Supplementary Figure 6a). Altogether, these data indicate that loss of PKM2 leads to reduced levels of SAM, in a manner that is independent of cell cycle arrest or regulation by P53. A reduction in the SAM:SAH ratio has been linked to a decrease in cellular methylation capacity^34^, and as a consequence, we identified a significant reduction in global DNA methylation, as assessed by measuring the levels of methylated cytosine in control and PKM2^KD^ ECs (Fig. 6c).

### Loss of PKM2 leads to endogenous retrovirus expression

Methylation of DNA controls a number of biological processes that include promoter inactivation, X-chromosome silencing and the silencing of transposable elements^35^. Retrotransposons make up close to 45% of the human genome^36^ and consist of two classes, long terminal repeat (LTR) and non-LTR transposons^37^. De-repression of these elements is associated with activation of the endogenous antiviral defence pathway^37^. In response to an increase of cytosolic viral RNA, a signalling cascade is triggered that begins with recognition by cytosolic sensors, activation of interferon regulatory factor (IRF)-NF-κB signalling and subsequent induction of interferon-stimulated gene (ISG) expression^38^. Intriguingly, global knockout of *Pkm2* in mouse was recently shown to increase systemic inflammation^14^, and *Pkm2*^*−/−*^ MEFs display increased expression of the ISGs *Ifi44* and *Ifih1*^25^, leading us to hypothesize that loss of PKM2 may lead to the activation of innate immune signalling as a consequence of reduced DNA methylation. Analysis of our proteomics data set revealed changes in the expression of genes associated with antiviral defence (Fig. 6d and Supplementary Figure 6b). Furthermore, mRNA levels of a number of ISGs were increased in PKM2^KD^ ECs (Supplementary Figure 6c), and analysis of published microarray data^25^ revealed that ISGs are induced in *Pkm2*^*−/−*^ MEFs (Supplementary Figure 6d), indicating that loss of PKM2 can lead to the activation of an innate immune response in multiple cell types and species. DNA methyltransferase (DNMT) inhibition was recently shown to permit the expression of endogenous retroviral (ERV) elements, leading to the formation of cytosolic dsRNA and activation of innate antiviral signalling^39,40^. *IFIH1* (MDA5) encodes a cytosolic pattern recognition receptor that recognizes double-stranded RNA (dsRNA)^41^, and its expression is upregulated in both PKM2 loss-of-function ECs (Supplementary Figure 6c) and MEFs (Supplementary Figure 6d). We therefore investigated if loss of PKM2 might lead to the accumulation of dsRNAs in ECs using the J2 antibody, and found a significant increase in dsRNA levels in PKM2^KD^ ECs (Fig. 6e, f). In addition, RT-qPCR analyses of control and PKM2^KD^ ECs revealed an increase in the expression of a subclass of ERVs (Fig. 6g), which are induced in response to DNMT inhibition^39^. To understand if increased ERV expression in PKM2^KD^ ECs was linked to changes in DNA methylation, we performed site-specific restriction digestion of PCR-amplified bisulfite-converted DNA at the *MLT1B* and *MER4D* loci. These analyses revealed a mild reduction in DNA methylation at the *MLT1B* and *MER4D* loci in PKM2^KD^ ECs (Fig. 6h), the extent of which was likely limited by the cell cycle arrest observed following loss of PKM2. Histone H3 lysine 9 trimethylation (H3K9me3), which has been shown to contribute to ERV repression in embryonic stem cells^42^, was not changed in PKM2^KD^ ECs (Supplementary Figure 7a). Collectively, our data indicate that loss of PKM2 leads to reduced DNA methylation, which triggers viral mimicry induced by expression of endogenous retroviral transcripts.

### ERV expression levels are limited by RELB

P53 has been shown to limit the expression of retrotransposons in MEFs treated with DNMT inhibitors^43^, and the NF-κB transcription factor family is known to induce the expression of ISGs, forming a critical component of the innate immune response to viral infection^44^. Given the increased expression of both P53 and RELB in the absence of PKM2, we hypothesized that these transcription factors may regulate ISG and/or ERV expression in ECs. Loss of P53 did not significantly alter ISG expression (Supplementary Figure 7b), ERV expression (Supplementary Figure 7b) or DNA methylation (Supplementary Figure 7c) in PKM2^KD^ ECs. In contrast, *RELB* silencing led to a dramatic increase in ERV and ISG mRNA levels in both RELB^KD^ and PKM2^KD^/RELB^KD^ ECs (Fig. 6i). Furthermore, analysis of previously published microarray data from *Relb*^*−/−*^ mouse bone-marrow-derived dendritic cells revealed increased expression of numerous ISGs (Supplementary Figure 7d)^45^, indicating that RELB limits innate immune activation in multiple cell types. RELB is known to suppress transcription through the directed recruitment of DNA methyltransferases and chromatin remodelling factors^46^, leading us to hypothesize that RELB may limit ERV expression by regulating local chromatin composition. Analysis of the *MLT1B* and *MER4D* loci showed that RELB^KD^ did not significantly alter DNA methylation (Supplementary Figure 7e), while H3K9me3 levels were also unchanged (Supplementary Figure 7f). Collectively, these data show that RELB is a key regulator of the innate immune response in ECs, which acts in a DNA methylation and H3K9me3-independent manner to limit ERV expression (Supplementary Figure 7g).

## Discussion

This study highlights the importance of PKM2 for growth and homoeostasis of the vascular endothelium and identifies molecular changes associated with its absence. PKM2 has been linked with anabolic cell growth in various experimental settings^10,11,47^, and its loss is associated with a PKM1-dependent impairment of pyrimidine synthesis and cell cycle arrest in MEFs^25^. In the present study, we observed distinct changes in metabolism in PKM2^KD^ and PKM^KD^ ECs, indicating that the PKM isoforms differentially regulate EC metabolism. For example, oxygen consumption and [U-13C_6_]-glucose labelling of citrate were increased only in PKM2^KD^ ECs, indicating that PKM1 may enhance the oxidative metabolism of glucose in mitochondria. In contrast, we observed reduced labelling of lactate and α-KG, and increased mitochondrial superoxide levels in PKM2^KD^ and PKM^KD^ ECs, indicating that the levels of these metabolites are predominantly regulated by PKM2. Recent genetic analyses in mouse have demonstrated that chronic deletion of PKM2 does not impact cell proliferation, due to compensatory upregulation of the *Pkm* splice isoform PKM1^14,48^. Our genetic analysis of PKM2 function, using conditional deletion of PKM2 specifically in ECs, reveals that loss of PKM2 reduces EC proliferation in vivo and indicates that acute loss or transient inhibition of PKM2 may lead to fundamentally different effects on cell growth than chronic loss. Furthermore, we provide evidence that impaired nucleotide synthesis in cells lacking PKM2 can be regulated at the transcriptional level by RELB-P53 signalling. The NF-κB and P53 transcriptional networks are known to respond to a variety of cellular stress stimuli, and our data indicate that RELB-P53 signalling may play an important role in the response of the endothelium to metabolic dysfunction, by limiting cell growth.

There is an increasing appreciation of the important link between intermediary metabolism and chromatin structure and function^32,49^, with the availability of nutrients linked to modifications of DNA^50^ and histones^51^. One of the most striking metabolic changes that we observed in ECs lacking PKM2 was a reduction in the one-carbon metabolite, SAM, which is required for the methylation of a broad range of substrates, including cytosine residues in DNA. In yeast, the PKM2 orthologue Pyk1 was identified as a component of a complex named SESAME (serine-responsive SAM containing metabolic enzyme complex), that was shown to regulate chromatin modifications^52^. While in the present study we did not identify an orthologous mammalian SESAME complex, we observed protein-level changes in a number of key enzymes in the serine, glycine and one-carbon metabolic network. Decreased expression of the SAM-synthesizing enzyme MAT2A and reduced steady-state levels of SAM were independent of P53, indicating that this effect is not merely a consequence of cell cycle arrest. Taken together, these data indicate that loss or inhibition of PKM2 may lead to changes in chromatin structure and function.

As a consequence of DNA hypomethylation, we observed increased ERV, dsRNA and ISG levels in ECs lacking PKM2. The fold induction of ERV and ISG expression in PKM2^KD^ ECs was more limited than previously reported in HCT116 cells treated with DNMT inhibitors^40^. These differences may reflect the relatively minor changes in DNA methylation observed in PKM2^KD^ ECs, or result from cell-type-specific differences in ERV de-repression, as previously described^53^. Examination of published microarray analyses of *Pkm2* knockout MEFs^25^ shows increased expression of a number of ISGs, indicating that loss of PKM2 can trigger innate immune signalling in different cell types. In addition to its role in the maintenance of metabolic homoeostasis, global deletion of *Pkm2* was recently shown to result in systemic inflammation in mice^14^. Our data indicate that inflammation associated with loss of PKM2 may, at least in part, be due to the de-repression of endogenous retroviral elements that trigger chronic activation of autoimmune signalling in both ECs and other cell types. More broadly, they indicate that metabolic dysfunction may be a trigger for autoimmune disease. The role of NF-κB in the regulation of immune signalling is well established^54^. We have uncovered a homoeostatic function for RELB as a suppressor of ERV expression in ECs, which limits activation of antiviral innate immune signalling. The mechanisms through which RELB limits ERV expression remain to be identified, and could involve modifications to local chromatin composition or direct regulation of transcription (Supplementary Figure 7g). Examination of previously published microarray analysis of bone marrow dendritic cells from *Relb*^*−/−*^ mice^45^ shows that RELB limits ISG expression, likely by limiting ERV expression, in multiple cell types. Furthermore, *Relb* knockout mice display chronic inflammation characterized by immune cell infiltration into multiple tissues^55^, a phenotype we hypothesize may occur as a result of ERV de-repression. Collectively, these findings broaden our understanding of how endothelial metabolism is controlled and link metabolic changes with the control of growth, epigenetics and immune responses in ECs.

## Methods

### Cell culture

Human umbilical vein endothelial cells (HUVECs) were purchased from Lonza or Life Technologies, authenticated for EC marker expression and cultured until the fourth passage. HUVECs were cultured in endothelial basal medium-2 (EBM-2, Lonza) supplemented with foetal bovine serum, hydrocortisone, human basic fibroblast growth factor, vascular endothelial growth factor, R3-insulin-like growth factor, ascorbic acid, human epidermal growth factor, GA-1000 and heparin. Human embryonic kidney (HEK) cells were cultured in Dulbecco’s modified Eagle’s medium (DMEM) supplemented with 10% foetal calf serum (Thermo), 100 units/ml penicillin and 100 μg/ml streptomycin. Both cell lines were cultured in 5% CO_2_ at 37 °C.

### RNA interference

For pan-PKM or PKM2-specific silencing, siRNAs directed against *PKM* or specifically targeting exon 10 of the *PKM* gene have been described previously^20^. We validated the effect of three siRNA duplexes by assessing knockdown efficiency using RT-qPCR and western blot, and cell proliferation by quantifying EdU^+^ cell numbers, glucose uptake, lactate and ATP levels. The following siRNA duplex was used for further studies: PKM2_si156:ccauaaucguccucaccaa. *P53*, *RELA*, *RELB* and *REL* were targeted with pools of siRNA duplexes (Mission esiRNA, Sigma). A negative control pool of four siRNAs (ON-TARGETplus Non-targeting pool, Dharmacon) was used to control for transfection. Transfections were performed using Lipofectamine RNAiMAX (Invitrogen) with the manufacturer's standard protocol. For all RNAi experiments, cells were re-seeded 24 h after siRNA transfection to reach a confluence of ~80% at 48 h after transfection.

### RT-qPCR

RNA was isolated using the miRNeasy micro Kit (Qiagen) combined with on-column DNase digestion (DNase-Free DNase Set, Qiagen). An aliquot of 1 μg of RNA was used for reverse transcription with the Maxima First Strand cDNA Synthesis Kit (Thermo). All PCR reactions were run on a CFX Connect Real-Time System (Biorad). The following primer pairs were used: *PKM1*_fwd-CTATCCTCTGGAGGCTGTGC, *PKM1*_rev-CCATGAGGTCTGTGGAGTGA; *PKM2*_fwd-CCACTTGCAATTATTTGAGGAA, *PKM2*_rev-GTGAGCAGACCTGCCAGACT; *TBP*_fwd-GGAGAGTTCTGGGATTGTAC, *TBP*_rev-CTTATCCTCATGATTACCGCAG. For analysis of endogenous retrovirus expression, previously published primer pairs were used^40^. Expression levels were normalized to *Tbp* expression and fold changes calculated using the ∆∆Ct method.

### Western blot analysis and antibodies

HUVECs were lysed in modified RIPA buffer (150 mM NaCl, 50 mM Tris–HCl pH 7.4, 1% IGEPAL, 0.1% sodium deoxycholate, 1 mM EDTA) supplemented with protease inhibitors (cOmplete ULTRA Mini, Roche) and PMSF. Proteins were separated using SDS-PAGE on precast TGX gradient gels (Biorad) and then transferred to polyvinylidine fluoride membranes using the Transblot Turbo Transfer System (Bio-Rad). Membranes were blocked in 5% BSA for 1 h, probed with primary antibodies overnight at 4 °C and then with peroxidase-conjugated secondary antibodies. The following antibodies were used: PKM1 (Cell Signaling Technology, #7067, 1:1000), PKM2 (Cell Signaling Technology, #4053, 1:1000), P21 (Cell Signaling Technology, #2947, 1:1000), P53 (Cell Signaling Technology, #9282, 1:500), P53 (Santa Cruz, sc-126, 1:1000), RELB (Cell Signaling Technology, #10544, 1:1000), MAT2A (Novus Biologicals, #NB110-94158, 1:2000), β-actin (Cell Signaling Technology, #8457, 1:1000), GAPDH (Cell Signaling Technology, #2118, 1:2000) and anti-rabbit IgG-HRP (Santa Cruz Biotechnology, sc-2004, 1:10,000). For analysis, membranes were incubated with ECL (Clarity Western ECL Substrate, Biorad) and imaged using a ChemiDoc MP system (Biorad). Uncropped images of blots are presented in Supplementary Figure 8.

### Seahorse metabolic analysis

Functional metabolic analyses in HUVECs were performed using a Seahorse XFe96 analyser (Seahorse Bioscience) according to the manufacturer’s protocol. In brief, HUVECs (40,000 per well) were seeded on fibronectin-coated XFe96 microplates. After 2 h, the cell medium was changed to a non-buffered assay medium and cells were maintained in a non-CO_2_ incubator for 1 h. Using the Glycolysis Stress Test Kit, extracellular acidification rate (ECAR) was assessed under basal conditions, and sequentially following the addition of glucose (10 mM), the mitochondrial ATP synthase inhibitor oligomycin (3 µM) or 2-deoxy-d-glucose (2-DG; 100 mM), an inhibitor of glycolysis. Using the Mito Stress Test Kit, oxygen consumption rate (OCR) was measured sequentially under basal conditions, after injection of oligomycin (3 µM), the mitochondrial uncoupler carbonyl cyanide-4-(trifluoromethoxy)phenyl-hydrazone (FCCP; 1 µM), and after injection of a mix of the respiratory chain inhibitors antimycin A (1.5 µM) and rotenone (3 µM).

### Lactate assay

Lactate concentration was measured in HUVEC cell culture medium that was conditioned for 12 h. The measurement was performed with a Lactate assay kit (Biovision) following the manufacturer’s protocol.

### Glucose uptake assay

Endothelial glucose uptake was assessed by analysing the uptake of 2-NBDG (2-(N-(7-Nitobenz-2oxa-1,3-diazol-4-yl)Amino)-2-Deoxyglucose) (Life Technologies), a fluorescent glucose analogue. HUVECs were incubated for 1 h in media containing 100 µM 2-NBDG and fluorescence was measured using the BD FACS LSR II flow cytometer. Data were analysed with the BD FACSDiva software (Version 8.0.1).

### Immunostaining of cell cultures

Twenty-four hours after transfection, HUVECs were re-seeded in 96-well imaging plates (Ibidi) to reach ~80% confluence at 48 h after transfection. For EdU staining, cells were cultured in EGM2 media containing 10 μM EdU (Thermo) for 3 h prior to fixation at 37 °C and in 5% CO_2_. Cells were fixed in 4% PFA for 15 min at room temperature and then permeabilised with 0.5% Triton X-100 for 15 min. To prevent antibody binding to non-specific epitopes, blocking was performed for 1 h in 5% goat serum, 1% BSA and 0.1% Triton X-100. Primary antibodies were diluted in blocking solution and incubated overnight at 4 °C. The following primary antibodies were used: phospho-Histone H3 (Cell Signaling Technology, #9713, 1:500) and J2A (Scicons, 1:200). Alexa Fluor-conjugated secondary antibodies were used for detection (Thermo, 1:250). The Click-iT EdU Alexa Fluor 647 imaging kit was used for detection of EdU incorporation (Thermo). Imaging was performed using an LSM800 Observer (Zeiss).

### Cell cycle analyses

Control, PKM2^KD^, PKM^KD^, P53^KD^ and/or PKM2^KD^/P53^KD^ HUVECs were harvested 48 h after transfection at ~80% confluence. Adherent cells were fixed in ice-cold 70% ethanol and then stored at −20 °C overnight. For staining, cells were washed twice in PBS, resuspended in a solution of 50 mg/ml propidium iodide (Sigma-Aldrich) containing RNase (Sigma-Aldrich #R6513) in 0.1% Triton X-100 and incubated for 20 min at 37 °C. Data were acquired using a BD FACS LSR II flow cytometer.

### In vitro scratch wound assay

HUVECs were transfected with contol or PKM2 siRNAs and after 24 h seeded in 24-well plates to reach 100% confluency at 48 h after transfection. At this stage, a scratch wound was applied to the EC monolayer using a 200 μl pipette tip, and individual wells were imaged (*T*_0_). HUVECs were incubated for a further 8 h in complete media, photographed again and the percentage wound closure in ctrl and PKM2^KD^ ECs calculated using ImageJ.

### Generation of zebrafish mutants with TALENs and CRISPR/Cas9

Animals were maintained under standard conditions and all experiments were conducted in accordance with institutional (MPG) and national ethical and animal welfare guidelines. TALENs were used to generate *pkma2*^*s717*^ mutants and CRISPR/Cas9 was used to generate *pkmb*^*s718*^ mutants as previously described^56^. For the generation of *pkma2* mutants, TALEN arms composed of the following RVDs targeting exon 10 of the *pkma* gene were used: NN NI HD HD HD NN NG NN NI HD HD HD NG NG HD NI NN NI NG NN HD and NG NG NN NI NI NN NN NI NI NN HD HD NG HD NN NI HD NN NN. RNA was synthesized with mMESSAGE mMachine (Ambion) and 50 pg per TALEN arm was injected into one-cell stage embryos to generate potential founders (*F*_0_). Analysis of mRNA expression in *pkma2*^*+/+*^ and *pkma2*^*−/−*^ embryos at 26 hpf revealed no changes in *pkma1* expression in *pkma2* mutants (Supplementary Figure 2h). For CRISPR/Cas9, sgRNAs were designed using the CRISPR Design online toolbox (http://crispr.mit.edu) and the following guide sequence was used to target exon 5 of the *pkmb* gene: TGTGGAACAGGGCGTGGACATGG. Guide RNAs were made by ligating annealed primers into the pT7-gRNA vector, and the pT3Ts-nCas9n plasmid (Addgene) was used to synthesize Cas9 mRNA. To generate founders, between 50 pg of sgRNA and 250 pg of Cas9 mRNA were coinjected into one-cell stage embryos. *F*_0_ founders for each gene were identified by outcrossing TALEN or CRISPR/Cas9-injected fish with AB and screening the offspring at 24 hpf using HRMA. PCR products from *F*_0_ founders that showed promising HRMA melt curves were cloned and sequenced, and those with frameshift mutations were grown. Primer sequences for HRMA genotyping were as follows: *pkma2*_HRMA_fwd: CTGTTCTGACCCGTGACCCTTC; *pkma2*_HRMA_rev: GGTGAGTATGATGATGCCGC; *pkmb*_HRMA_fwd: CTGAGAAGGACATCAAGGACC; *pkmb*_HRMA_rev: AGCAGCTTTGCGGATAAAGGAGG. Mutants were generated in the *Tg(kdrl:EGFP)*^*s843*^ background^57^.

### Mouse experiments

Animals were maintained under standard conditions and all experiments were conducted in accordance with institutional (MPG) and national ethical and animal welfare guidelines. Conditional PKM2 mutant mice (*Pkm*^*tm1.1Mgvh*^) were backcrossed to the C57/Bl6 background. For conditional EC-specific deletion, PKM2-floxed mice were bred with *Tg(Pdgfb-icre/ERT2)*^1*Frut*^ mice, which express the tamoxifen-inducible CreERT2 in ECs. For analysis of angiogenesis in the retina, postnatal mice were injected intraperitoneally with 25 μl of 4-Hydoxytamoxifen (2 mg/ml, H6278, Sigma) on postnatal days (P)1–P3 and retinas were harvested on P7. Control animals were littermates without CreERT2 expression. Due to the nature of the experimental setup, animals were randomly assigned to treatment groups. Only litters that reached normal body weight at P7 were used. Mice that were more than 1.5 interquartile ranges (IQR) above or below the upper or lower IQR for their respective litter were not analysed.

### Immunostaining and analysis of postnatal retinas

For quantitative analysis of angiogenesis, eyes were harvested from P7 mice and fixed in 4% PFA on ice for 2 h. After fixation, eyes were washed in PBS and then dissected to remove the retinal tissue. Following dissection, retinas were incubated in blocking buffer (3% normal goat serum, 1% BSA, 0.5% Triton X-100 and 0.5% Tween-20) for 1 h at RT and then incubated with primary antibodies diluted in incubation buffer (1.5% normal goat serum, 0.5% BSA, 0.25% Triton X-100 and 0.25% Tween-20) overnight at 4 °C. Primary antibodies against the following proteins were used: PECAM (1:200, #AF3628, R&D Systems), ERG (1:200, #ab92513, Abcam), Collagen IV (1:400, #2150-1470, AbD Serotec). After primary antibody incubation, retinas were washed four times in 0.1% PBS Triton X-100 and incubated with Alexa Fluor-conjugated secondary antibodies (Thermo, 1:250) for 2 h at RT. Following secondary antibody staining, retinas were washed four times in 0.1% PBS Triton X-100 and the Click-iT EdU Alexa Fluor 647 imaging kit was used for detection of EdU incorporation (Thermo). Retinas were mounted on glass slides using Vectashield (Vector Labs) and imaging was performed using a Zeiss LSM700 or Leica SP8 microscope. All images were acquired without prior knowledge of the genotype of the individual samples.

### Quantitative analysis of the retinal vasculature

Outgrowth of the retinal vasculature was measured using ImageJ software as the distance from the centre of the optic nerve to the periphery of the vessel bed in each leaflet of dissected retinas. The mean distance of individual leaflets was taken as the value for each sample. For quantification of EC and EdU^+^ EC number, vessel area, branch point density and empty Collagen IV sleeves, four fields per sample were acquired using a ×25 objective. The total number of ECs and EdU^+^ EC number was determined using Imaris Software. The mean of four fields per sample at the angiogenic front or central plexus was taken as the value for each sample. EC area per field and branch point density was determined using the AngioTool software. Empty Collagen IV sleeves were counted manually using ImageJ. These analyses were not blinded.

### LC-HRMS metabolomics and flux analysis

For measurement of steady-state metabolite levels, control, PKM2^KD^, PKM^KD^, P53^KD^ and/or PKM2^KD^/P53^KD^ HUVECs were harvested 48 h after transfection in 80% methanol pre-cooled to −80 °C. Plates were transferred to a −80 °C freezer for 15 min to inactivate enzyme functions, then scraped into extraction solvent and centrifuged at 20,000 × *g* for 10 min at 4 °C. The supernatant was then collected and dried for ~2 h in a speed-vac at room temperature. Dried pellets were stored at −80 °C. For flux analyses, 50:50 labelling was performed using [U-13C_6_]glucose (Cambridge Isotope Laboratories, Inc) as a tracer, and metabolite extraction and storage was performed as above. Metabolomics was performed using liquid chromatography-high resolution mass spectrometry (LC-HRMS). An ultimate 3000 UHPLC (Dionex) is coupled to the Q Exactive Plus-Mass spectrometer (QE-MS, Thermo Scientific, San Jose, CA) for metabolite separation and detection. Detailed instrument method information was described previously^58^. Samples were reconstituted into 30 µl sample solvent (water:methanol:acetonitrile, 2:1:1, v/v), and 4 μl was injected to the LC-QE-MS. The peak area of each metabolite was used to present relative abundance and to calculate the isotopologue distribution. All data are provided in Supplementary Data File 1.

### Proteomics analysis

Control and PKM2^KD^ HUVECs were harvested in 4% SDS, 0.1 M Tris–HCl (pH 7.6) 48 h after transfection. Samples were sonicated, clarified by centrifugation and protein concentration was estimated using the DCA protein assay kit. Proteins were separated using SDS-PAGE on precast TGX gradient gels (Bio-Rad) and stained using the Colloidal Blue staining kit (Invitrogen). Each lane of the gel was cut into slices, and proteins were digested as previously described^56^. Briefly, gel pieces were washed with 50 mM ammoniumbicarbonate and ethanol, reduced with 10 mM DTT for 45 min at 56 °C, carbamidomethylated with 55 mM iodoacetamide for 30 min at RT in the dark, and then digested with trypsin overnight. The next day, peptides were extracted from the gel pieces using increasing concentrations of acetonitrile. Samples were cleaned up with Stage Tips (Stop and go extraction) and then measured with LC-MS/MS on a QExactive HF. Separation of peptides according to their hydrophobicities was achieved on a 15 cm in-house packed column (internal diameter 75 μm, C18 Beads (Dr. Maisch) diameter 1.8 μm) using a binary buffer system: (A) 0.1% formic acid in H_2_O and (B) 0.1% formic acid in 80% acetonitrile. The following gradient of 75 min was used: 5 min 10% of B, 50 min 38% of B, 5 min 60% of B, 10 min 95% of B, 5 min 5% of B. Mass spectra were acquired at a resolution of 60,000 (200 *m/z*) using an AGC target of 3e6 and a maximal injection time of 20 ms. A top 15 method was applied for subsequent acquisition of high-energy collision-induced dissociation (HCD) fragmentation MS/MS spectra of the 15 most intense peaks. Fragmentation (MS/MS) was performed with a resolution of 15,000 at 2.2 *m/z* and and AGC target of 1e5 within a maximal injection time of 25 ms. Raw files were processed using MaxQuant 1.5.5.1 and the Andromeda search engine. For peptide assignment, MS/MS HCD fragmentation spectra were correlated to the Uniprot Homo sapiens database (2016). Further downstream analyses were performed as previously described. All data are provided in Supplementary Data File 2.

### RNA-seq analysis and data processing

RNA was isolated from 48 h after transfection from control, P53^KD^, PKM2^KD^ and PKM2^KD^/P53^KD^ HUVECs using the miRNeasy micro Kit (Qiagen) combined with on-column DNase digestion (DNase-Free DNase Set, Qiagen). RNA and library preparation integrity were verified with a BioAnalyzer 2100 (Agilent) or LabChip Gx Touch 24 (Perkin Elmer).2 µg of total RNA was used as input for Truseq Stranded mRNA Library preparation following the low sample protocol (Illumina). Sequencing was performed on the NextSeq500 instrument (Illumina) using v2 chemistry, resulting in a minimum of 37M reads per library with 1 × 75 bp single end setup. The resulting raw reads were assessed for quality, adaptor content and duplication rates with FastQC^59^. Reaper version 13–100 was employed to trim reads after a quality drop below a mean of Q20 in a window of ten nucleotides^60^. Only reads between 30 and 150 nucleotides were cleared for further analyses. Trimmed and filtered reads were aligned to the GRCh37.p5 human genome assembly using STAR 2.4.0a with the parameter “--outFilterMismatchNoverLmax 0.1” to increase the maximum ratio of mismatches to mapped length to 10%^61^. The number of reads aligning to genes was counted with the featureCounts 1.4.5-p1 tool from the Subread package^62^. Only reads mapping at least partially inside exons were admitted and aggregated per gene. Reads overlapping multiple genes or aligning to multiple regions were excluded. Differentially expressed genes were identified using DESeq2 version 1.62^63^.

### 5-methylcytosine quantification

The levels of 5-methylcytosine were measured using the MethylFlash Methylated DNA quantification kit (Epigenetik) according to the instructions of the manufacturer.

### Chromatin immunoprecipitation (ChIP)

ChIP was performed using the truChIP Chromatin Shearing Reagent kit (Covaris) according to the manufacturer’s instructions. Chromatin was sheared (Bioruptor, Diagenode) to generate fragments between 200 and 400 bp. 5 μg of DNA was used for each immunoprecipitation and 10% was stored as an input. Immunoprecipitation was performed following a published protocol^64^ using the following antibodies: Mouse IgG (2 μg/IP, 5441S, Cell Signaling) and Histone H3 (tri methyl K9) (2 μg/IP, ab8898, Abcam). Following immunoprecipitation and reverse cross-linking, samples and inputs were purified using the NucleoSpin Gel and PCR Clean-up kit (Macherey-Nagel) following the manufacturer’s instructions for samples containing SDS.

### Bisulfite conversion and restriction analyses

Genomic DNA was isolated from 80% confluent control, PKM2^KD^, P53^KD^, PKM2^KD^/P53^KD^, RELB^KD^ and PKM2^KD^/RELB^KD^ HUVECs 48 h after transfection, using the DNeasy Blood & Tissue kit (Qiagen, 69504). Bisulfite conversion was performed using the EpiJET Bisulfite Conversion Kit (Thermo Scientific, #K1461) according to the manufacturer’s instructions. PCR amplification of bisulfite-converted DNA was performed using Phusion U Hot Start DNA Polymerase (Thermo Scientific, F555S) with the following primers: *MLT1B*_Fwd—AGTTATAGATAGGTATAGAGTGTTGGTTTATAG; *MLT1B*_Rev—CCTATTCTAACCATTTTCTATCAATAAAATC; *MER4D*_Fwd—TTTGAGGAAGTGTGTTTGAGGTTGTTAGGATG; *MER4D*_Rev—CCTCTAACACTACCTTAACTCAAACATTTC. Following PCR amplification, samples were purified and then digested with HpyCH4III (*MER4D*) or HincII (*MLT1B*) for 8 h, and visualized on a 3% agarose gel.

### Blinding

The individual genotypes of mice and zebrafish were unknown before data analysis was performed.

### Statistical analyses

No statistical tests were used to predetermine sample size. Statistical analyses were performed by unpaired, two-tailed Student's *t* test, or non-parametric one-way ANOVA followed by Tukeys HSD test using GraphPad Prism software. Data are represented as mean ± s.d. for individual data points and mean ± s.e.m. where values presented are calculated as a mean of means. *P* values <0.05 were considered significant. All metabolomic (Figs. 1c–l, 4b, 5f, 6b, and Supplementary Figures 1e–j, 5f–g, 6a), transcriptomic (Figs. 4c, 6d and Supplementary Figures 4a, e, 5a, 6b), proteomic (Fig. 5e and Supplementary Figures 5b, 6c, 7b), RT-qPCR (Figs. 1a, 6g, i and Supplementary Figures 1c, 7b), ChIP RT-qPCR (Supplementary Figure 7a, f) and phenotypic (Figs. 2a–c, 5a, c and Supplementary Figures 1a-c, 5c, d) analyses were performed by transfecting primary HUVECs pooled from multiple donors (*n* = number of independent transfections). For in vivo analyses in zebrafish (Fig. 2d, e) and mouse (Fig. 3b, d and Supplementary Figure 3e), *n* = number of independent animals observed. Sample sizes were selected on the basis of published protocols^65^.

## Electronic supplementary material

Supplementary Information

Description of Additional Supplementary Files

Supplementary Movie 1

Supplementary Data 1

Supplementary Data 2

Supplementary Data 3

## Data Availability

RNA-seq data are deposited in the Gene Expression Omnibus under accession number GSE84877. Metabolomics (Supplementary Data File 1) and proteomics (Supplementary Data File 2) data are provided as supplementary data files.
