# Supplementary material for: Loss of pyruvate kinase M2 limits growth and triggers innate immune signaling in endothelial cells
Source: Nat Commun. 2018 Oct 9;9:4077. doi: 10.1038/s41467-018-06406-8 (PMC6177464; doi:10.1038/s41467-018-06406-8)
Supplement: Supplementary file 1 — Supplementary Information [file 41467_2018_6406_MOESM1_ESM.pdf]

Supplementary Information

Loss of Pyruvate Kinase M2 limits growth and triggers innate immune signaling in endothelial cells.

Stone et al.

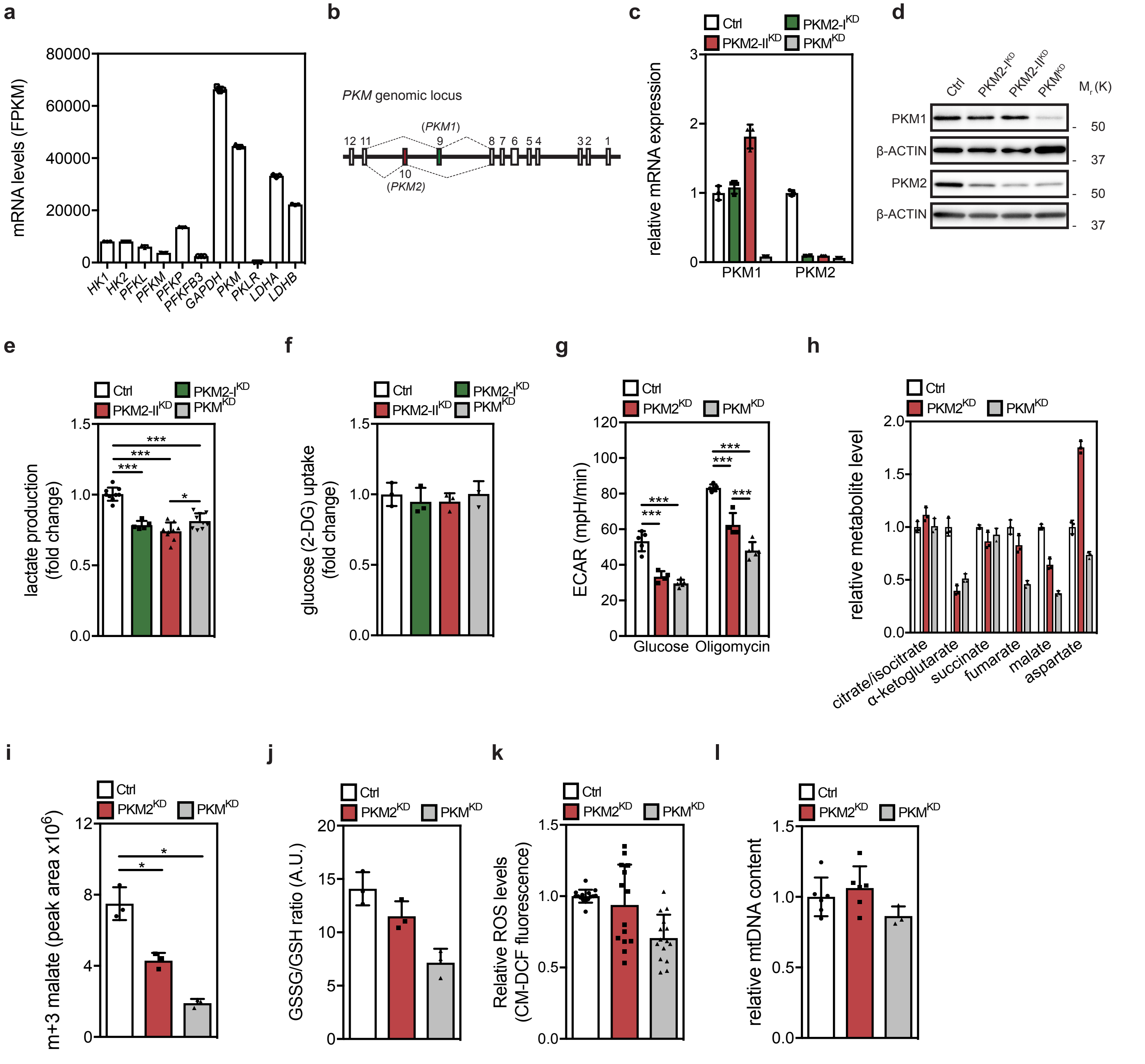

Supplementary Figure 1: PKM2 silencing alters mitochondrial metabolism.
(**a**) mRNA levels for selected glycolytic enzymes in HUVECs (RNA-seq analyses on control HUVECs (FPKM - fragments per kilobase of transcript per million mapped reads)). (**b**) Schematic representation illustrating alternative splicing of the human *PKM* gene, leading to the inclusion of either exon 9 (*PKM1*) or exon 10 (*PKM2*). siRNA duplexes were used to specifically target *PKM2* mRNA (exon 10) or all *PKM* transcripts (exon 4, leading to the silencing of *PKM1* and *PKM2* expression). (**c**) RT-qPCR analysis of *PKM1* and *PKM2* mRNA expression showing specific knockdown of the relevant transcripts using siRNA duplexes specific to *PKM2* or pan-*PKM* in ECs (n=3). (**d**) Western blot analysis of PKM1 and PKM2 expression in HUVECs treated with the indicated siRNA duplexes. (**e**) Relative lactate levels in control, PKM2-I^{KD}, PKM2-II^{KD} and PKM^{KD} ECs (n=9). (**f**) Glucose uptake in control, PKM2-I^{KD}, PKM2-II^{KD} and PKM^{KD} ECs (n=3). (**g**) Extracellular acidification rate (ECAR) in cells treated with or without oligomycin, showing basal and maximal glycolytic rate in control, PKM2^{KD} and PKM^{KD} ECs (n=5). (**h**) Relative levels of TCA cycle intermediates in control, PKM2^{KD} and PKM^{KD} ECs (n=3). (**i**) Incorporation of [U-13C$_6$]-glucose into m+3 malate in control, PKM2^{KD} and PKM^{KD} ECs (n=3). (**j**) Ratio of glutathione to oxidized glutathione in control, PKM2^{KD} and PKM^{KD} ECs (n=3). (**k**) Relative ROS levels in control, PKM2^{KD} and PKM^{KD} ECs (n=14). (**l**) Relative mitochondrial DNA content in control, PKM2^{KD} and PKM^{KD} ECs (n=3). **a**, **c**, **e-l** data represent means ± s.d. (***$P < 0.001$, *$P < 0.05$ by one-way analysis of variance (ANOVA) followed by Tukey's HSD test).

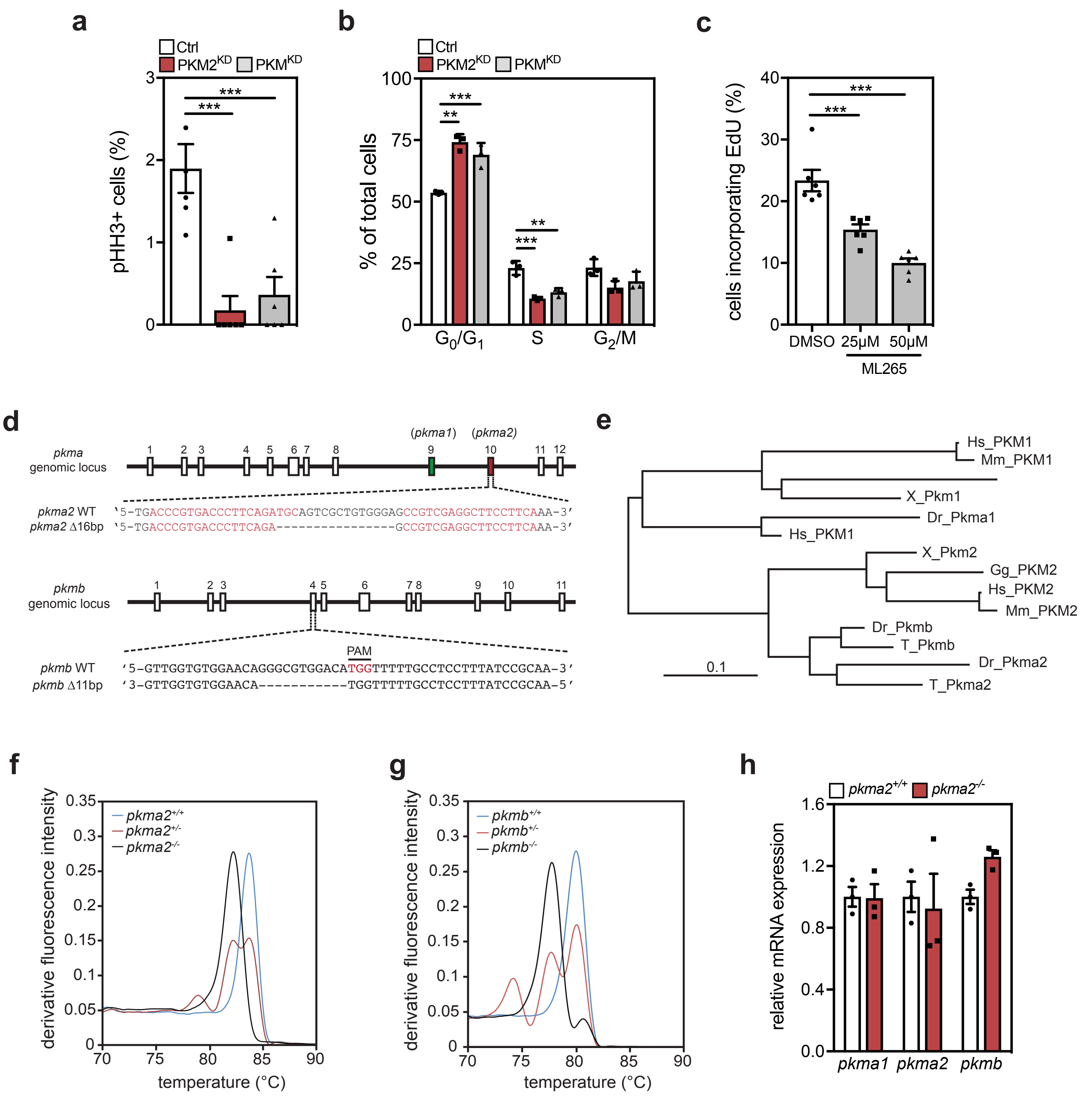

Supplementary Figure 2: *In vitro* analysis of endothelial growth and zebrafish loss-of-function models. (**a**) Quantification of phospho-Histone H3 positive cell numbers in control, $PKM2^{KD}$ and PKM^{KD} ECs (n=6). (**b**) Flow cytometry analysis of cell cycle in propidium iodide stained control, $PKM2^{KD}$ and PKM^{KD} ECs (n=3). (**c**) Quantification of EdU positive cell numbers in DMSO and ML265 treated ECs (n=6). (**d**) Genomic loci of the zebrafish *pkma* and *pkmb* genes, sequences targeted using TALEN (*pkma2*) or CRISPR (*pkmb*) and the recovered lesions. (**e**) Clustering analysis based on a 56 amino acid sequence from each of the indicated proteins, which displays homology to the amino acids encoded by either exon 9 or 10 of the human *PKM* gene (Hs - Human, Mm - Mouse, X - *Xenopus*, Dr - Zebrafish, Gg - Chicken). High resolution melt analysis (HRMA) genotyping of *pkma2* (**f**) and *pkmb* (**g**) mutant zebrafish alleles. (**h**) mRNA expression levels of zebrafish *pkm* transcripts in *pkma2*$^{+/+}$ *and pkma2*$^{-/-}$ embryos at 26 hpf. **a,c, h** data represent means ± s.e.m. **b** data represent means ± s.d. (***P < 0.001, **P < 0.01 by one-way analysis of variance (ANOVA) followed by Tukey's HSD test).

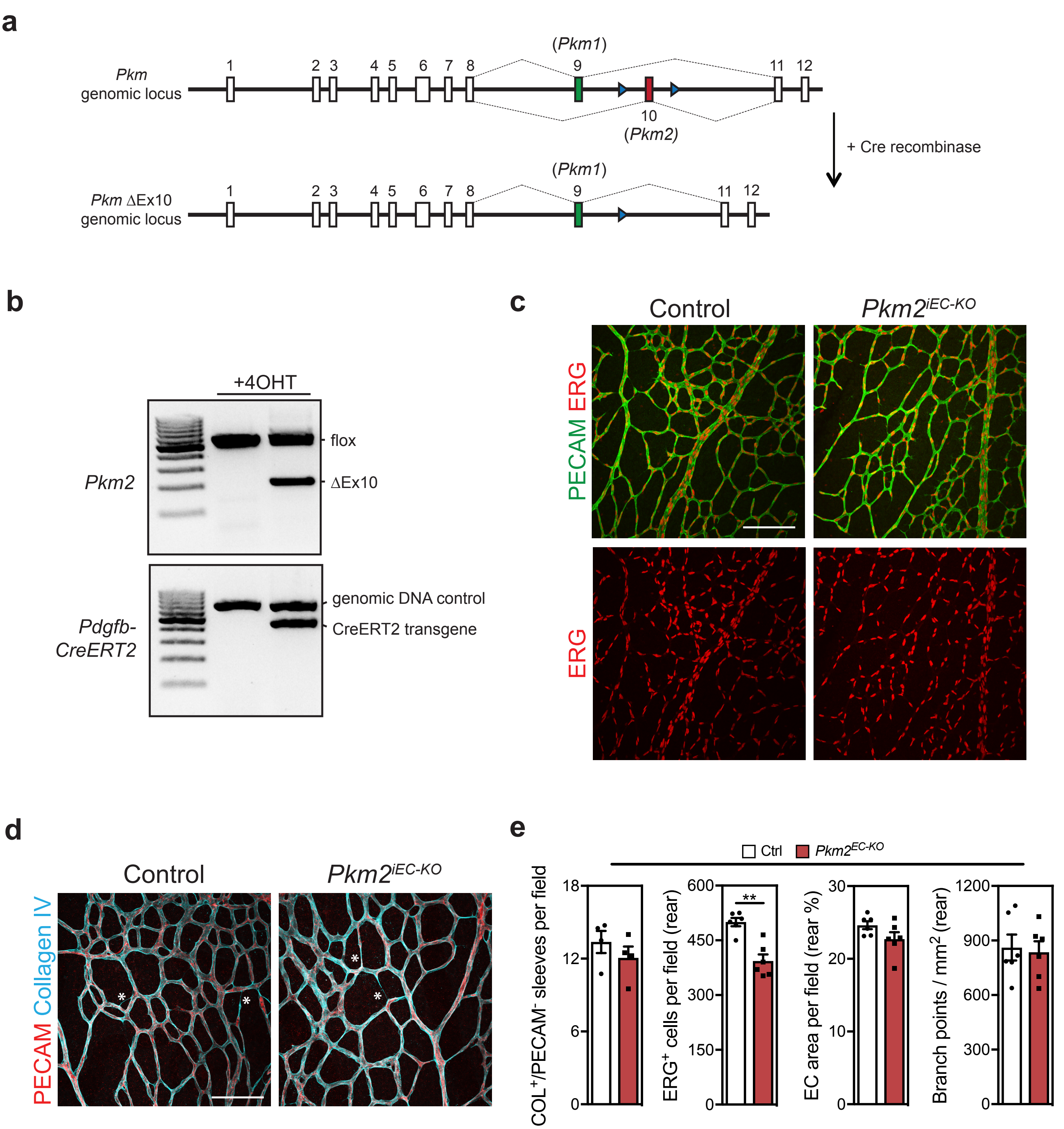

Supplementary Figure 3: Endothelial specific deletion of *Pkm2* in the post-natal mouse retina.
(**a**) Schematic representation illustrating alternative splicing of the mouse *Pkm* gene, leading to the inclusion of either exon 9 (*Pkm1*) or exon 10 (*Pkm2*), blue arrows indicate loxP sites in $Pkm^{tm1.1Mgvh}$ mutant mice and below is a schematic representation of the recombined locus. (**b**) PCR from genomic DNA of P7 $Pkm2^{fl/fl}$ mice showing efficient 4-OHT induced recombination of exon 10 from the *Pkm* gene in mice carrying (right) the *Pdgfb-CreERT2* transgene.
(**c**) Representative confocal projections of central plexus vessels in PECAM and ERG stained retinas from control and $Pkm2^{iEC-KO}$ mice. (**d**) Representative confocal projections of central plexus vessels in PECAM and Collagen IV stained retinas from control and $Pkm2^{iEC-KO}$ mice. (**e**) Quantification of Collagen IV^+/$PECAM^+$ sleeves, ERG^+ ECs, EC area per field and branch point density at the angiogenic front of control and $Pkm2^{iEC-KO}$ mice (n=6). **e** data represent means ± s.e.m. (**P < 0.01 by by two tailed students t-test) Scale bars in **c** and **d** = 100µm.

a

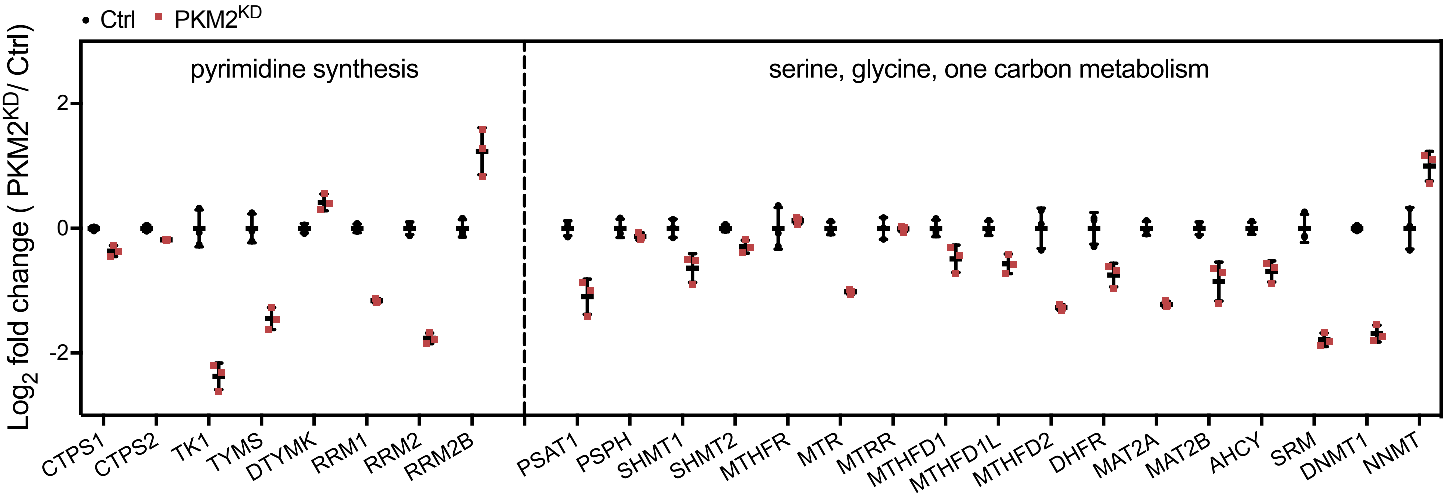

Supplementary Figure 4: Loss of PKM2 is associated with coordinated protein expression changes.
(**a**) Relative protein expression levels of enzymes in the pyrimidine synthesis and serine, glycine, one-carbon metabolism pathways in control and PKM2^{KD} ECs (related to heatmap in Figure 4c). **a** data represent means ± s.d.

Supplementary Figure 5

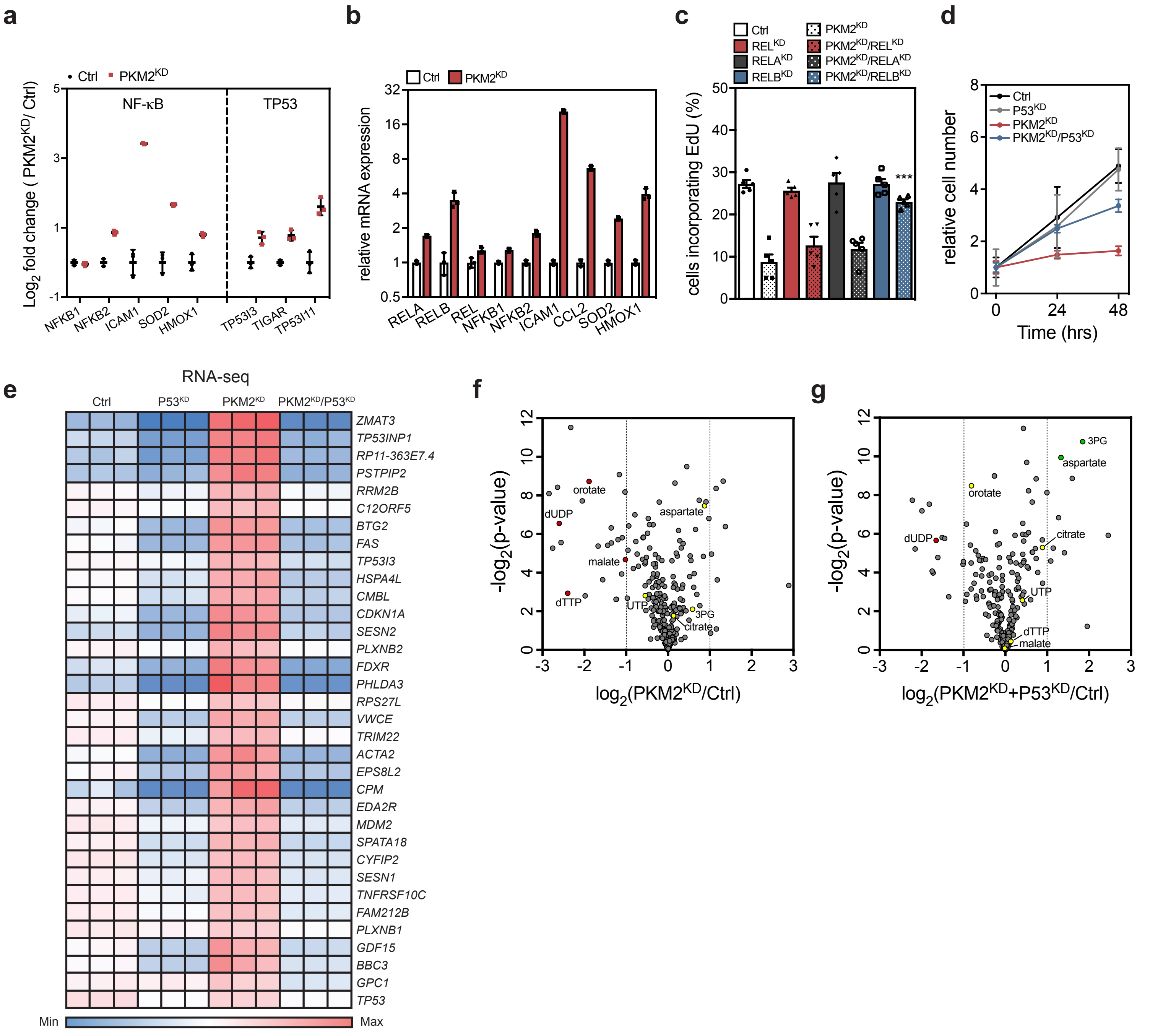

Supplementary Figure 5: RELB-P53 signalling limits proliferation of primary endothelial cells. (**a**) Relative protein expression levels of components of the NF-κB and P53 pathways in control and PKM2^{KD} ECs. (**b**) mRNA expression levels of NF-κB transcription factors and selected targets in control and PKM2^{KD} ECs (RT-qPCR, n=3). (**c**) EdU positive cell numbers in control, PKM2^{KD}, REL^{KD}, PKM2^{KD}/REL^{KD}, RELA^{KD}, PKM2^{KD}/RELA^{KD}, RELB^{KD} and PKM2^{KD}/RELB^{KD} ECs (n=6). (**d**) Relative cell numbers at the indicated time points in control, P53^{KD}, PKM2^{KD} and PKM2^{KD}/P53^{KD} ECs (n=6). (**e**) Heatmap of P53 target gene expression changes in control, P53^{KD}, PKM2^{KD} and PKM2^{KD}/P53^{KD} ECs. Metabolomics profile of (**f**) control vs PKM2^{KD} and (**g**) control vs PKM2^{KD}/P53^{KD} ECs (log$_2$(fold change) vs -log$_2$(p-value), two tailed students t-test, n=3). **a**,**b** data represent means ± s.d., **c**,**d** data represent means ± s.e.m. (***P < 0.001 by one-way analysis of variance (ANOVA) followed by Tukey's HSD test).

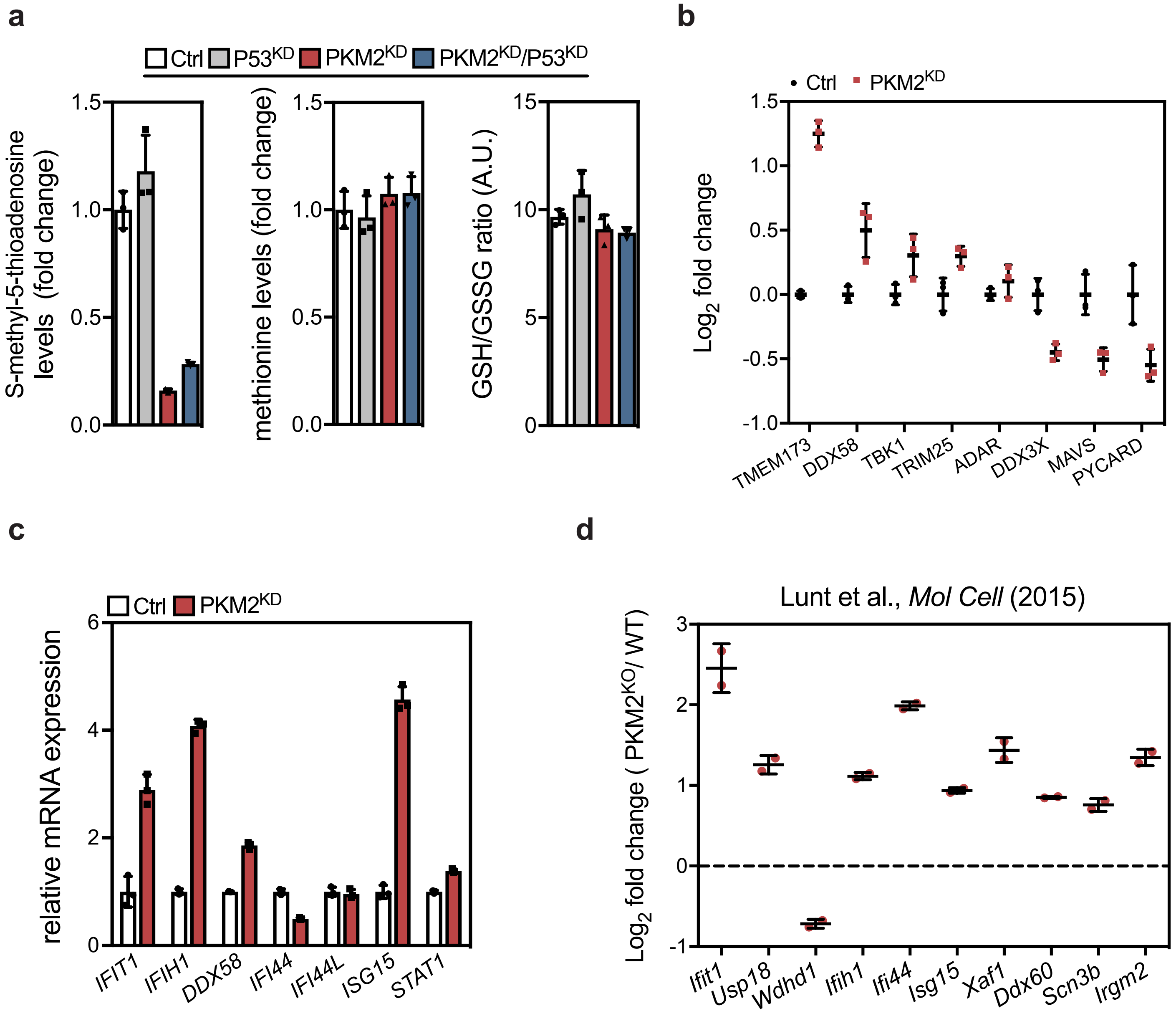

Supplementary Figure 6: Loss of PKM2 leads to activation of innate antiviral signalling.
(**a**) Relative levels of S-methyl-5-thioadenosine, methionine and GSH/GSSG ratio in control, $P53^{KD}$, $PKM2^{KD}$ and $PKM2^{KD}/P53^{KD}$ ECs (n=3). (**b**) Protein expression levels of viral responsive genes in control and $PKM2^{KD}$ ECs (n=3). (**c**) mRNA expression levels of interferon stimulated genes in control and $PKM2^{KD}$ ECs (RNA-seq analysis, n=3). (**d**) mRNA expression levels of the 10 most statistically significantly changed genes from wild type and *Pkm2*$^{-/-}$ mouse embryonic fibroblasts (microarray analysis, n=2, re-analysed from Lunt et al., 2015). **a**-**d**, data represent means ± s.d.

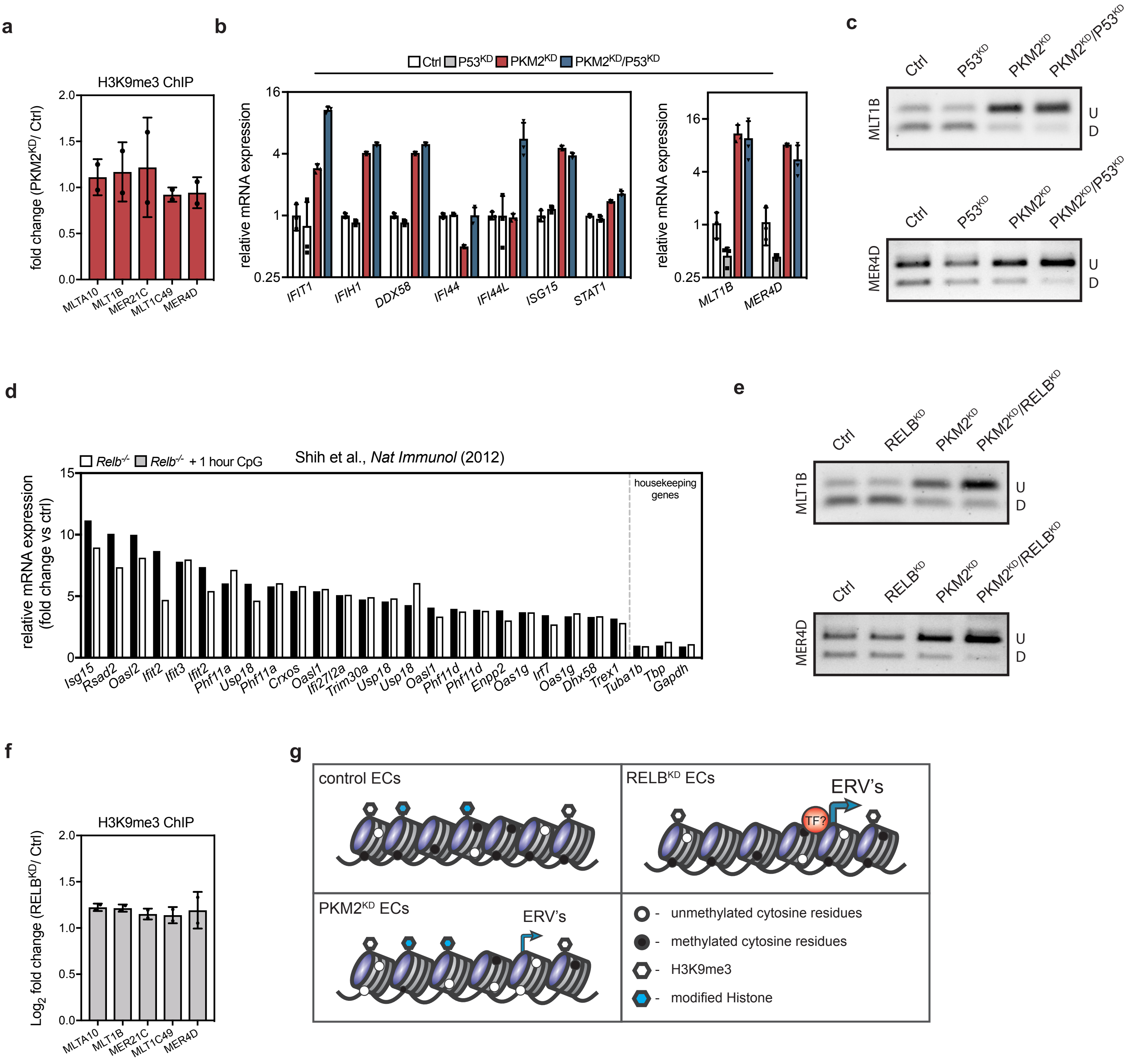

Supplementary Figure 7: PKM2 and RELB limit endogenous retrovirus expression and innate immune signalling in endothelial cells.
(**a**) RT-qPCR analysis at the indicated loci of H3K9me3 chromatin immunoprecipitated DNA from control and PKM2^{KD} ECs. (**b**) mRNA expression levels of interferon stimulated genes in control , P53^{KD}, PKM2^{KD} and PKM2^{KD}/P53^{KD} ECs (RNA-seq analysis, n=3) and relative mRNA expression of the indicated endogenous retroviruses in control, P53^{KD}, PKM2^{KD} and PKM2^{KD}/P53^{KD} ECs (n=3). (**c**) Restriction digestion of bisulfite-treated DNA amplified from the MLT1B and MER4D genom-ic loci from control, P53^{KD}, PKM2^{KD} and PKM2^{KD}/P53^{KD} ECs (U, undigested/unmethylated DNA; D - digested/ methylated DNA). (**d**) Relative mRNA expression of the top 25 most regulated probes in a microarray analysis of wild-type versus *Relb*$^{-/-}$ dendritic cells treated with or without CpG for 1 hour (data from Shih et al., 2012). (**e**) Restriction digestion of bisulfite-treated DNA amplified from the *MLT1B* and *MER4D* genomic loci from control, RELB^{KD}, PKM2^{KD} and PKM2^{KD}/ RELB^{KD} ECs
(U, undigested/unmethylated DNA; D - digested/methylated DNA). (**f**) RT-qPCR analysis at the indicated loci of H3K9me3 chromatin immunoprecipitated DNA from control and RELB^{KD} ECs. (**g**) Proposed model of DNA methylation and endogenous retrovirus expression changes in control, PKM2^{KD} and RELB^{KD} ECs. **a**, **b** and **f**, data represent means ± s.d. (***P < 0.001, **P < 0.01, *P < 0.05 by one-way analysis of variance (ANOVA) followed by Tukey's HSD test).

Supplementary Figure 8

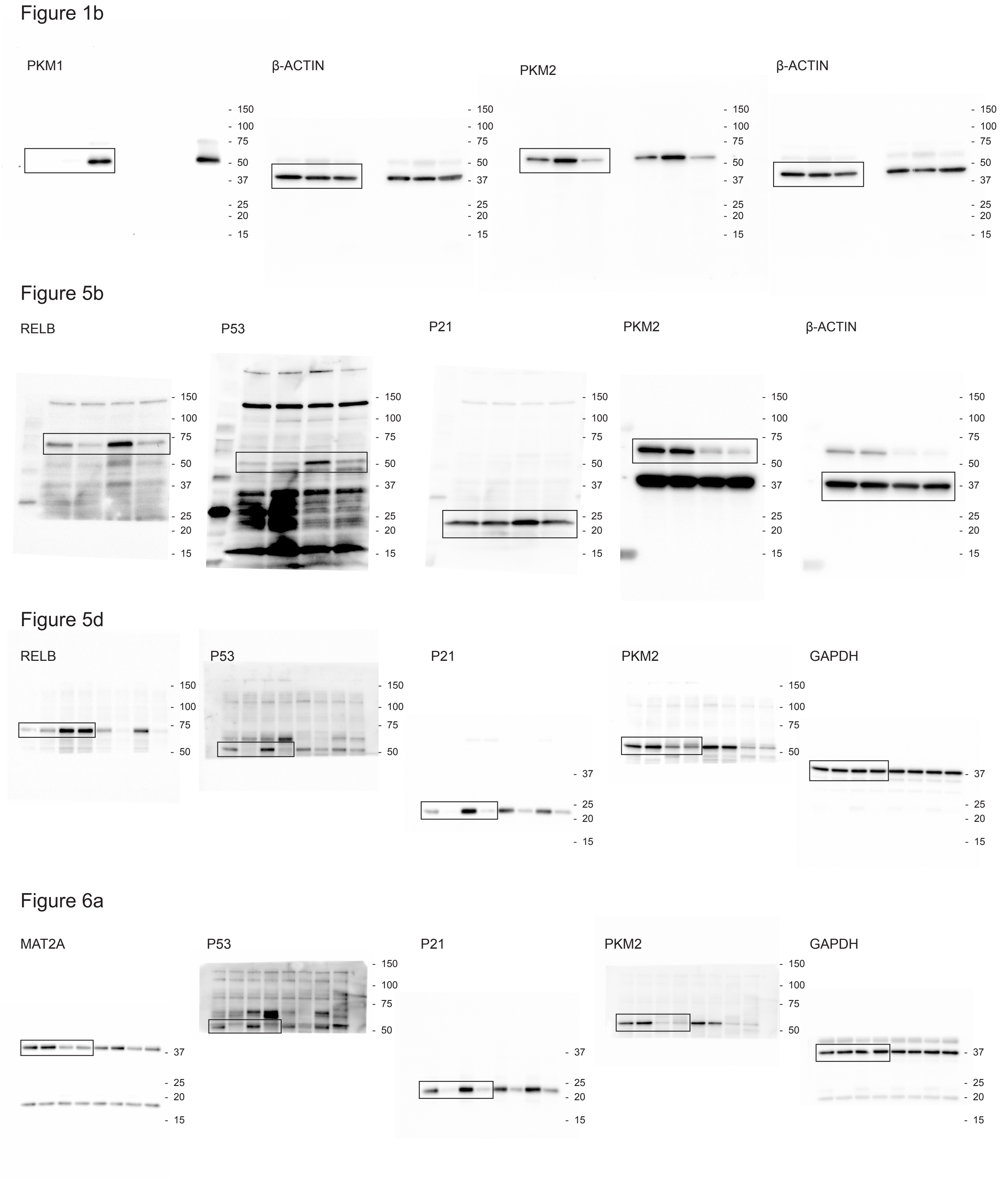

Supplementary Figure 8: Uncropped images relating to indicated Western blot data.
