# Supplementary material for: Loss of pyruvate kinase M2 limits growth and triggers innate immune signaling in endothelial cells
Source: Nat Commun. 2018 Oct 9;9:4077. doi: 10.1038/s41467-018-06406-8 (PMC6177464; doi:10.1038/s41467-018-06406-8)
Supplement: Supplementary file 2 — Description of Additional Supplementary Files [file 41467_2018_6406_MOESM2_ESM.pdf]

Description of Additional Supplementary Files

File Name: Supplementary Movie 1
Description: Live imaging of ISV angiogenesis in *pkma2^{mz}/pkmb$^{-/-}$* mutant zebrafish. Time-lapse confocal movie of a *Tg(kdrl:EGFP) pkma2^{mz}/pkmb$^{-/-}$* embryo between 26 and 48 hpf.

File Name: Supplementary Data 1
Description: Steady-state and [U-13C_6]glucose metabolomics data for HUVEC treated with siRNAs targeting Scr/PKM2/PKM or Scr/P53/PKM2/PKM2+P53

File Name: Supplementary Data 2
Description: Proteomics data for HUVEC treated with siRNAs targeting Scr/PKM2.

File Name: Supplementary Data 3
Description: Sequences of primers used in this study
